# The Role of Transient Receptor Potential A1 and G Protein-Coupled Receptor 39 in Zinc-Mediated Acute and Chronic Itch in Mice

**DOI:** 10.3389/fnmol.2021.768731

**Published:** 2022-01-12

**Authors:** Yue Hu, Qing-Yue Fu, Dan-Ni Fu, Xue-Long Wang, Zhi-Hong Wang, Jiang-Tao Zhang, Wen-Jing Xu, Guo-Kun Zhou, Li-Hua Chen, Tong Liu

**Affiliations:** ^1^Jiangsu Key Laboratory of Neuropsychiatric Diseases and Institute of Neuroscience, Soochow University, Suzhou, China; ^2^Department of Thoracic Surgery, Capital Medical University Electric Power Teaching Hospital Beijing, Beijing, China; ^3^Institute of Pain Medicine and Special Environmental Medicine, Nantong University, Nantong, China; ^4^Department of Nutrition and Food Hygiene, School of Public Health, Nantong University, Nantong, China; ^5^College of Life Sciences, Yanan University, Yan'an, China; ^6^Suzhou Key Laboratory of Intelligent Medicine and Equipment, Soochow University, Suzhou, China

**Keywords:** Itch, Zn^2+^, TRPA1, GPR39, p-ERK

## Abstract

Itching is a common symptom of many skin or systemic diseases and has a negative impact on the quality of life. Zinc, one of the most important trace elements in an organism, plays an important role in the regulation of pain. Whether and how zinc regulates itching is largely unclear. Herein, we explored the role of Zn^2+^ in the regulation of acute and chronic itch in mice. It is found that intradermal injection (i.d.) of Zn^2+^ dose-dependently induced acute itch and transient receptor potential A1 (TRPA1) participated in Zn^2+^-induced acute itch in mice. Moreover, the pharmacological analysis showed the involvement of histamine, mast cells, opioid receptors, and capsaicin-sensitive C-fibers in Zn^2+^-induced acute itch in mice. Systemic administration of Zn^2+^ chelators, such as N,N,N′,N′-Tetrakis(2-pyridylmethyl)ethylenediamine (TPEN), pyrithione, and clioquinol were able to attenuate both acute itch and dry skin-induced chronic itch in mice. Quantitative polymerase chain reaction (Q-PCR) analysis showed that the messenger RNA (mRNA) expression levels of zinc transporters (ZIPs and ZnTs) significantly changed in the dorsal root ganglia (DRG) under dry skin-induced chronic itch condition in mice. Activation of extracellular signal-regulated kinase (ERK) pathway was induced in the DRG and skin by the administration of zinc or under dry skin condition, which was inhibited by systemic administration of Zn^2+^ chelators. Finally, we found that the expression of GPR39 (a zinc-sensing GPCR) was significantly upregulated in the dry skin mice model and involved in the pathogenesis of chronic itch. Together, these results indicated that the TRPA1/GPR39/ERK axis mediated the zinc-induced itch and, thus, targeting zinc signaling may be a promising strategy for anti-itch therapy.

## Introduction

Itching (pruritus) is a common somatic sensation that is distinct from other senses, such as temperature, touch, and pain (Davidson and Giesler, 2010). It can lead to characteristic scratching behavior in mammals, even lip-rubbing behavior in zebrafish (Han and Simon, 2011). According to the duration, itching is divided into acute itch and chronic itch. Acute itch lasts several minutes to days and serves as an alarm system to remove the potential harmful stimulation from the body (Pfab et al., 2012). In contrast, chronic itch lasts for more than 6 weeks and is a common symptom of many diseases (Sakai et al., 2016; Andersen et al., 2017), including skin diseases, cholestasis, chronic kidney diseases (Mettang and Kremer, 2015; Cheng et al., 2019; Patel et al., 2019), neurological diseases (Misery et al., 2014), some cancer, and mental illness (Misery et al., 2018). Identification of novel itch mediators and related signaling pathways is not only helpful for understanding the mechanisms underlying chronic itch but may also lead to developing new effective anti-itch therapies.

Itch stimuli are detected by the peripheral nerve terminals of primary sensory neurons located in the dorsal root ganglia (DRG) and trigeminal ganglia (TG). The central branches of sensory neurons are responsible for transmitting peripheral itch signal to the dorsal horn of the spinal cord or spinal trigeminal nucleus (Dong and Dong, 2018), and then to the brain. Many pruritogens elicit itch through the activation of peripheral perceptive primary afferents, which are equipped with multiple receptors and/or ion channels for transducing itch signaling (Dong and Dong, 2018). Amounts of evidence showed that transient receptor potential (TRP) channels played a key role in the signal transduction process of itch sensation, including TRPV1, TRPA1, TRPV4, and TRPC4 (Moore et al., 2018). For example, TRPV1 mediated histamine-induced itch, which mainly involved histamine H1 and H4 receptors (Shim and Oh, 2008). TRPA1 was well-known for mediating histamine-independent itch, including mas-related G protein-coupled receptors (Mrgprs)-mediated itch (Wilson et al., 2011), oxidative stress-induced itch (Liu and Ji, 2012; Zhou et al., 2017), endothelin 1 (ET-1)-induced itch (Magnusdottir et al., 2020), miRNA-711-mediated cancer itch (Han et al., 2018), and 5-HT_7_ receptor-mediated serotonergic itch (Morita et al., 2015). Intriguingly, TRPA1 activation also played a critical role in multiple chronic itch conditions, including dry skin-induced chronic itch (Wilson et al., 2013), bile acids receptor TGR5-mediated cholestatic itch (Lieu et al., 2014), low-dose formalin-induced itch (Liu et al., 2021), methylglyoxal (MGO)-mediated diabetic itch (Cheng et al., 2019), tacrolimus-induced contact dermatitis pruritus (Wong et al., 2018), and imiquimod-induced psoriatic itch (Liu et al., 2010). Thus, targeting multiple TRP channels may be promising for developing a novel anti-itch therapy.

Zinc is the second most abundant trace metal element in the human body (after iron) (Carstens et al., 2020), and it is the only metal element that acts as a cofactor for more than 300 enzymes (Misery et al., 2014). As a biologically essential trace element, zinc is very important for cell growth, development, differentiation, senescence, apoptosis, homeostasis, DNA synthesis, and RNA transcription (Haider and Bhutta, 2009; Lin et al., 2017). Usually, zinc is present in the cell in the form of divalent cations, namely Zn^2+^ (Chasapis et al., 2012). Intracellular zinc homeostasis is strictly controlled by multiple zinc transporters and metallothioneins (MTs). The zinc transporters are divided into two families, including Zrt-and Irt-like proteins (ZIPs) and Zn transporters (ZnTs) (Kambe et al., 2014). The ZIPs family is encoded by the *SLC39As* and has 14 family members (ZIP1-ZIP14). They have 8 transmembrane domains that mediate the transport of Zn^2+^ from the extracellular organelles or vesicles to the cytoplasm (Prasad, 2013). The ZnTs family is encoded by the *SLC30As* and has 10 members (ZnT1-ZnT10) (Roohani et al., 2013; Hennigar and McClung, 2018). The MTs, widely distributed in the cytoplasm, have been proven to be donors and receptors of transcription factors and metalloenzymes (Bafaro et al., 2017; Si and Lang, 2018). The ZIPs, ZnTs, and MTs jointly regulate the transport, storage, and release of intracellular Zn^2+^ in order to maintain intracellular zinc homeostasis (Kambe et al., 2014). In addition, a plasma membrane G-protein coupled receptor (GPCR), called zinc sensing receptor/G-protein coupled receptor 39 (ZnR/GPR39) is responsible for sensing extracellular Zn^2+^ (Kashiv et al., 2016; Lu et al., 2016). Extracellular Zn^2+^ is used as a signal molecule to trigger intracellular Ca^2+^ signals through the activation of specific Gαq coupling GPR39 (Colvin et al., 2010; Atrian and Capdevila, 2013). The GPR39 can be activated not only by exogenous Zn^2+^ but also by endogenous Zn^2+^ released from the neuronal vesicles, the salivary gland vesicles, or intestinal epithelial cells under physiological and/or pathological conditions (Myers et al., 2017; Baltaci and Yuce, 2018; Thokala et al., 2019). To date, whether and how Zn^2+^ regulates itch remains largely unknown.

In the present study, we aimed to identify the molecular mechanism underlying Zn^2+^-mediated acute and chronic itch in mice. We found the Zn^2+^ dose-dependently induced acute itch in mice. We verified that TRPA1 (but not TRPV1 and TRPV4) participated in Zn^2+^-induced itch in mice. Activation of the extracellular signal-regulated kinase (ERK) was also involved in acute itch induced by Zn^2+^ in mice. Moreover, Zn^2+^ chelators, such as N,N,N′,N′-Tetrakis(2-pyridylmethyl)ethylenediamine (TPEN), pyrithione, and clioquinol, significantly attenuated acute and dry skin-induced chronic itch in mice. The Q-PCR analysis showed that the mRNA expression levels of ZIPs and ZnTs changed in the DRG of mice under dry skin condition. Moreover, TRPA1 and p-ERK participated in Zn^2+^-mediated chronic itch in mice. Thus, we demonstrated that the zinc/TRPA1/GPR39 axis played a critical role in Zn^2+^-induced acute and chronic itch in mice.

## Materials and Methods

### Animals

Male ICR mice and C57BL/6J mice (6–8 weeks old) were purchased from the Shanghai SLAC Laboratory Animal Co., Ltd. (Shanghai, China). Male *Trpa*1^−/−^and *Trpv*1^−/−^ mice were obtained from Jackson Laboratories (Bar Harbor, ME, USA). *Trpv*4^−/−^ mice were produced by Cam-Su Genomic Resource Center, Soochow University. All animals were kept on a 12-h light/dark cycle with free access to food and water, and the rooms were maintained at 22 ± 2°C and 40–60% humidity. All animal experiments were conducted in accordance with the National Institutes of Health Guide for the Care and Use of Laboratory Animals and were approved by the Experimental Animal Care and Use Committee of Soochow University.

### Neck Model of Acute Itch

According to the previous studies (Liu et al., 2012; Miao et al., 2018), mice were shaved at the nape of the neck more than 2 days before experiments. On the day of behavioral testing, the mice were placed in separate small plastic chambers (for mice: 10 × 10 × 12.5 cm^3^) on an elevated metal mesh floor for at least 40 min for habituation. Under brief anesthesia with isoflurane, saline, ZnCl_2_ (1–150 mM), Zn(CH_3_COO)_2_ (0.3–150 mM), ZnSO_4_ (1-150 mM), compound 48/80 (100 μg), chloroquine (200 μg), chlorpheniramine (300 μg), HC030031 (50, 100 μg), A967079 (50 μg), capsazepine (50 μg), HC067047 (50 μg), and TC-G-1008 (10–100 μg) were injected intradermally into the neck of mice through a 26G needle. The volume of the i.d. into the nape of the neck was 50 μl. Immediately after the injection, the mice were put back to the chambers and video-recorded for 30 min (Sony HDRCX610, Shanghai, China). The video was then replayed offline and scratching behavior was quantified in a blinded manner. One scratching bout was defined as lifting a hind leg from the ground and scratching the skin behind the ears or on the back and then placing the paw back on the ground.

### Cheek Model

The cheek model (Shimada and LaMotte, 2008) was employed to distinguish the itch and pain behaviors in mice. We shaved the cheek of mice more than 2 days before the experiment. One day after shaving, the mice were placed in small plastic chambers (10 × 10 × 12.5 cm^3^) on an elevated metal mesh floor and allowed at least 30 min to habituate. After brief anesthesia with isoflurane, the mice were given an intradermal injection of capsaicin (10 μg) and ZnCl_2_ (3 and 30 mM) into the cheek. The volume of the i.d. injection into the cheek was 10 μl. The mice were immediately placed back to the chambers after injection and recorded for 30 min (Sony HDRCX610, Shanghai, China). The video was subsequently replayed offline, scratching behavior and wiping behaviors were quantified in a blinded manner. We counted scratch bouts and wiping behaviors, respectively. The wiping behavior means the mice raising a forelimb toward the cheek over 1 s or a few seconds, then keeping their forelimb down.

### Dry Skin Chronic Itch Model

As previously reported (Jing et al., 2018), a dry skin model was established to induce chronic itch, by applying acetone and ether (1:1), followed by water (AEW), on the neck or cheek skin two times a day (9:00 a.m. and 4:00 p.m.) for 7 days. The spontaneous scratching was video recorded for 1 h in the 0th, 1st, 3rd, 5th, and 7th days and the total number of scratches was counted in a blinded manner.

### Western Blotting

About 10 or 30 min after i.d. injection of 3 mM ZnCl_2_, the mice were transcardially perfused with sterile saline under anesthesia with isoflurane. The DRG and the neck skin of the mice were rapidly isolated and homogenized in a lysis buffer containing a cocktail of phosphatase inhibitors and protease inhibitors for total protein extraction assays. The protein concentrations were measured by Pierce bicinchoninic acid (BCA) protein assay (Thermo); then equal amounts of protein (40 μg) were loaded onto each lane and separated on 10% sodium dodecyl-sulfate polyacrylamide gel electrophoresis (SDS-PAGE). After transfer, the membranes were blocked with 5% non-fat milk in Tris-HCl buffer saline (TBS) at room temperature for 1 h and then the polyvinylidene fluoride (PVDF) membranes were incubated overnight at 4°C with primary monoclonal anti-p-ERK (mouse, 1:1000; Santa Cruz Biotechnology, CA) and β-Tubulin (mouse, 1:2000, Novus). The blots were washed and incubated with horseradish peroxidase-conjugated goat anti-mouse IgG secondary antibody (1:2000, Vazyme). Protein bands were visualized using an enhanced chemiluminescence detection kit (Pierce) and the band densities were assessed and analyzed with NIH ImageJ software (NIH, Bethesda, MD).

### Real-Time Quantitative PCR

Total RNA from DRG and skin were extracted using trizol reagent (Invitrogen, MA, USA) according to the specifications of the manufacturer. The RNA was treated with DNase I (Invitrogen, MA, USA), and the complementary DNA (cDNA) was synthesized using a ThermoScript RT-PCR System kit (Invitrogen, MA, USA). Reactions were carried out in a volume of 10 μl per reaction containing 5 μl SYBR Green master mix (2×) (Cat#mf015, mei5bio; Beijing, China), 3 μl cDNA, and 2 μl primer mix using Opticon real-time PCR Detection System (ABI Life7500, Applied Biosystems, CA, USA). The list of primers used in this study is provided in detail in Supplementary Table 2. Relative mRNA expression levels of different target genes compared to glyceraldehyde-3-phosphate dehydrogenase (GAPDH) were calculated using 2^ΔΔC_T_^ methods.

### Hematoxylin and Eosin Staining

The skin was dissected immediately after the mice were killed and the tissues were postfixed in 4% paraformaldehyde overnight and the skin sections were cut (10 μm) in a cryostat. The sections were stained with hematoxylin and eosin (H&E). The stained sections were then dried, cleared, and covered for observation and photomicrography. The H&E staining technique was used to detect the epidermal thickness of the dry skin-induced chronic itch model.

### Von Frey Test

The mechanical pain threshold of mice was measured by Von Frey in this study (Chaplan et al., 1994). Von Frey filaments are a series of fibers with different lengths and diameters. When the filaments touches the sole of the mouse, it will produce a certain pressure. When the pressure rises to a certain level, the mouse will have a positive reaction. In this experiment, the Von Frey cilia have a series of cilia, which correspond to 0.07, 0.16, 0.4, 0.6, 1.0, 1.4, and 2.0 g, respectively. The bottom of the hind paws was poked according to the number of grams from small to large. On noticing the raising of the foot, licking the bottom of the foot, or throwing the foot, the response to the gram was recorded at once. Each stimulus was given 10 times. When there were 5 or more positive reactions, the number of grams at this time was counted as the mechanical pain threshold of the mouse.

### Open Field Test

In order to evaluate the locomotion of mice in the open field, the mice were placed alone in the central area of 40 ^*^ 40 cm in an open-air field with light. The bottom is divided into 4 × 4 grids, 16 squares of equal size (10^*^10 cm). Mice were allowed to explore the open field for 10 min. A video tracking software (ANY-maze) was used to count the track and distance of the mice.

### Rotarod Test

The motor ability was evaluated by the Rotarod system (ZH-300, Zhenghua Co. Ltd., China) in the rotarod test. The whole test included 4 days. On the first day, the mice were trained to keep them at a basic rotational speed of 4 rpm for 5 min without falling. On the second day, the mice were trained to rotate the rod from 4 to 20 rpm within 5 min to maintain balance without falling, and repeated on the third day. On the last day, the rotating rod program was adjusted to increase from 4 to 40 rpm within 5 min and the time on the rod was recorded.

### Bioinformatics Analysis

We acquired the normalized expression level of zinc transporter-related genes and itch-related genes from the “http://mousebrain.org/genesearch.html” website in each DRG subtypes. It was drawn by the heat map function in the software with GraphPad prism 8.

### Drugs and Administration

The chemicals, such as ZnCl_2_ (Cat#10023818), Zn(CH_3_COO)_2_ (Cat#30192618), and ZnSO_4_ (Cat#10024018) were obtained from Sinopharm Chemical Reagent Co, Ltd. (Shanghai, China). Compounds, 48/80 (Cat#C2313) and chloroquine (Cat#C6628) were obtained from Sigma-Aldrich (St. Louis, MO, USA). Pyrithione (Cat# HY-B1747-10 mg) and Clioquinol (Cat# HY-14603) were obtained from MCE (MedChemExpress, New Jersey, USA). TPEN (Cat#4309/100), HC030031 (Cat#2896), A967079 (Cat#4716), capsazepine (CPZ, Cat#0464), HC067047 (Cat#4100), and U0126 (Cat#U120) were obtained from Tocris (Bristol, UK). Morphine hydrochloride was obtained from China Northeast Pharmaceutical Group Shenyang No. 1 Pharmaceutical CO., Ltd (Shenyang City, Liaoning Province, China). Naloxone hydrochloride was obtained from China Sinopharm Group Guorui Pharmaceutical CO., Ltd (Huainan City, Anhui Province, China). TPEN, pyrithione, and clioquinol were dissolved in 10% dimethyl sulfoxide (DMSO). Unless otherwise specified, other reagents are dissolved in sterile saline.

### Statistical Analysis

Data were analyzed using Graphpad Prism 6.1 (GraphPad, La Jollar, CA, USA). All data were expressed as the mean ± SEM. Unpaired Student's *t*-test was used to compare the two groups. One-way ANOVA followed by *post-hoc* Bonferroni's test was used for multiple comparisons. Two-way ANOVA followed by *post-hoc* Bonferroni's test was used to analyze the data with repeated-measure over a time course. Differences were considered statistically significant at *P* < 0.05.

## Results

### Zn^2+^ Induced Dose-Dependent Scratching Behavior in Mice

**Figure 1:**
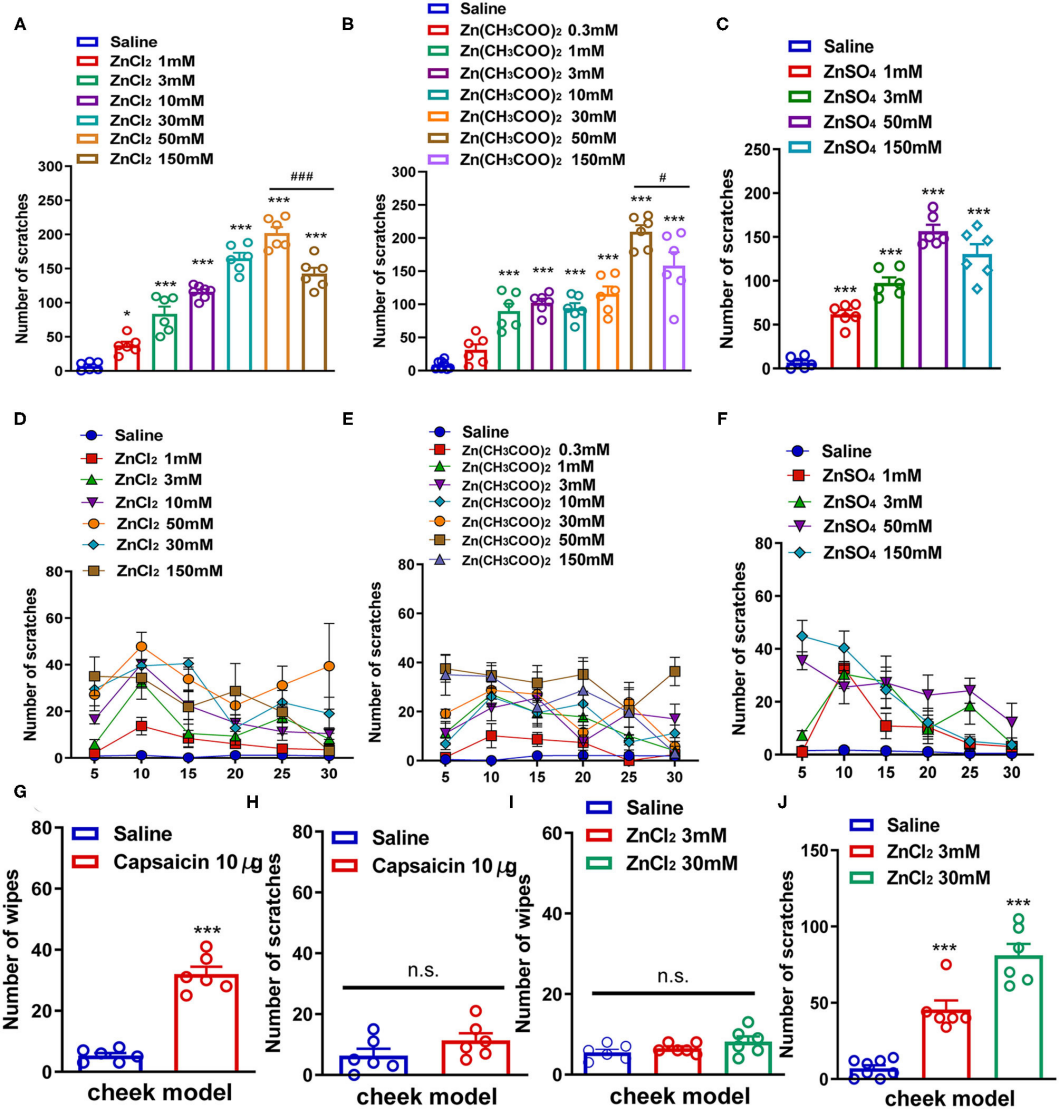
Zn^2+^ evoked a scratching behavior in the neck and cheek models of mice. **(A,D)** The total number **(A)** and time course **(D)** of the scratching behavior induced by intradermal (i.d.) injection of ZnCl_2_ (1–150 mM) in the nape of the neck in mice. **(B,E)** The total number **(B)** and time course **(E)** of scratching behavior induced by i.d. injection of Zn(CH_3_COO)_2_ (0.3–150 mM) in the nape of the neck in mice. **(C,F)** The total number **(C)** and time course **(F)** of the scratching behavior induced by i.d. injection of ZnSO_4_ (1–150 mM) in the nape of the neck in mice (*P < 0.05, ***P < 0.001 vs. Saline, ^###^*P* < 0.001 vs. 50 mM ZnCl_2_, ^#^*P* < 0.05 vs. 50 mM Zn(CH_3_COO)_2_, one-way AVOVA following *post-hoc* Bonferroni's test; *n* = 6–8 per group). **(G,H)** The total number of wiping **(G)** and scratching behavior **(H)** induced by i.d. injection capsaicin (10 μg) in the cheek in mice. **(I,J)** The total number of wiping **(I)** and scratching behavior **(J)** induced by i.d. injection ZnCl_2_ (3 and 30 mM) in the cheek in mice (***P < 0.001 vs. Saline, unpaired Student's *t*-test; *n* = 6–8 per group). All data are expressed by means ± SEM. n.s., not significant.

First, we used a neck model of acute itch to investigate whether i.d. injection of Zn^2+^ could induce the scratching behavior in mice by using three kinds of zinc compounds. ZnCl_2_ (1–150 mM), Zn(CH_3_COO)_2_ (0.3–150 mM), and ZnSO_4_ (1–150 mM) were injected intradermally into the neck of the mice. We found that i.d. injection of ZnCl_2_ (1–150 mM) in the nape of the neck evoked a scratching behavior in a dose-dependent manner in mice [*F*_(6,36)_ = 90.11, *P* < 0.0001; Figures 1A,D]. ZnCl_2_ began to evoke scratching at the dosage of 1 mM and reached a peak at the dosage of 50 mM. Interestingly, the highest dose of ZnCl_2_ (150 mM) significantly induced less scratches than that of 50 mM ZnCl_2_ (*t*_10_ = 4.714, *P* = 0.0008; Figure 1A). In addition, i.d. injection of Zn(CH_3_COO)_2_ (0.3–150 mM) into the nape of the neck also evoked scratching behavior in a dose-dependent manner in mice [*F*_(7,42)_ = 40.73, *P* < 0.0001; Figures 1B,E]. The Zn(CH_3_COO)_2_ began to evoke scratching at 1 mM and reached a peak at the dosage of 50 mM. However, the highest dose of Zn(CH_3_COO)_2_ (150 mM) induced significantly fewer scratches than that of Zn(CH_3_COO)_2_ (50 mM) (*t*_10_ = 2.283, *P* = 0.0456; Figure 1B). Moreover, i.d. injection of ZnSO_4_ (1–150 mM) into the nape of the neck evoked a scratching behavior in a dose-dependent manner in mice [*F*_(4,25)_ = 70.36, *P* < 0.0001; Figures 1C,F]. Thus, these data indicated that i.d. injection of Zn^2+^ was sufficient to induce scratching behavior in mice.

Subsequently, we established a mouse cheek model to further explore whether the scratching behavior induced by Zn^2+^ was itch-indicative or pain-indicative. The i.d. injection of capsaicin (10 μg) into the cheek only induced pain-indicative wiping (*t*_10_ = 10.40, *P* < 0.0001; Figure 1G) but not itch-indicative scratching in mice (*t*_10_ = 1.515, *P* = 0.1607; Figure 1H). The i.d. injection of ZnCl_2_ (3 and 30 mM) into the cheek of mice only induced itch-indicative scratching [*F*_(2,17)_ = 53.36, *P* < 0.0001] but not pain-indicative wiping [*F*_(2,15)_ = 2.154, *P* = 0.1506; Figures 1I,J].

### Histamine, Mast Cells, Opioid Receptors, and C-Fibers Were Involved in Zn^2+^-Induced Acute Itch in Mice

**Figure 2:**
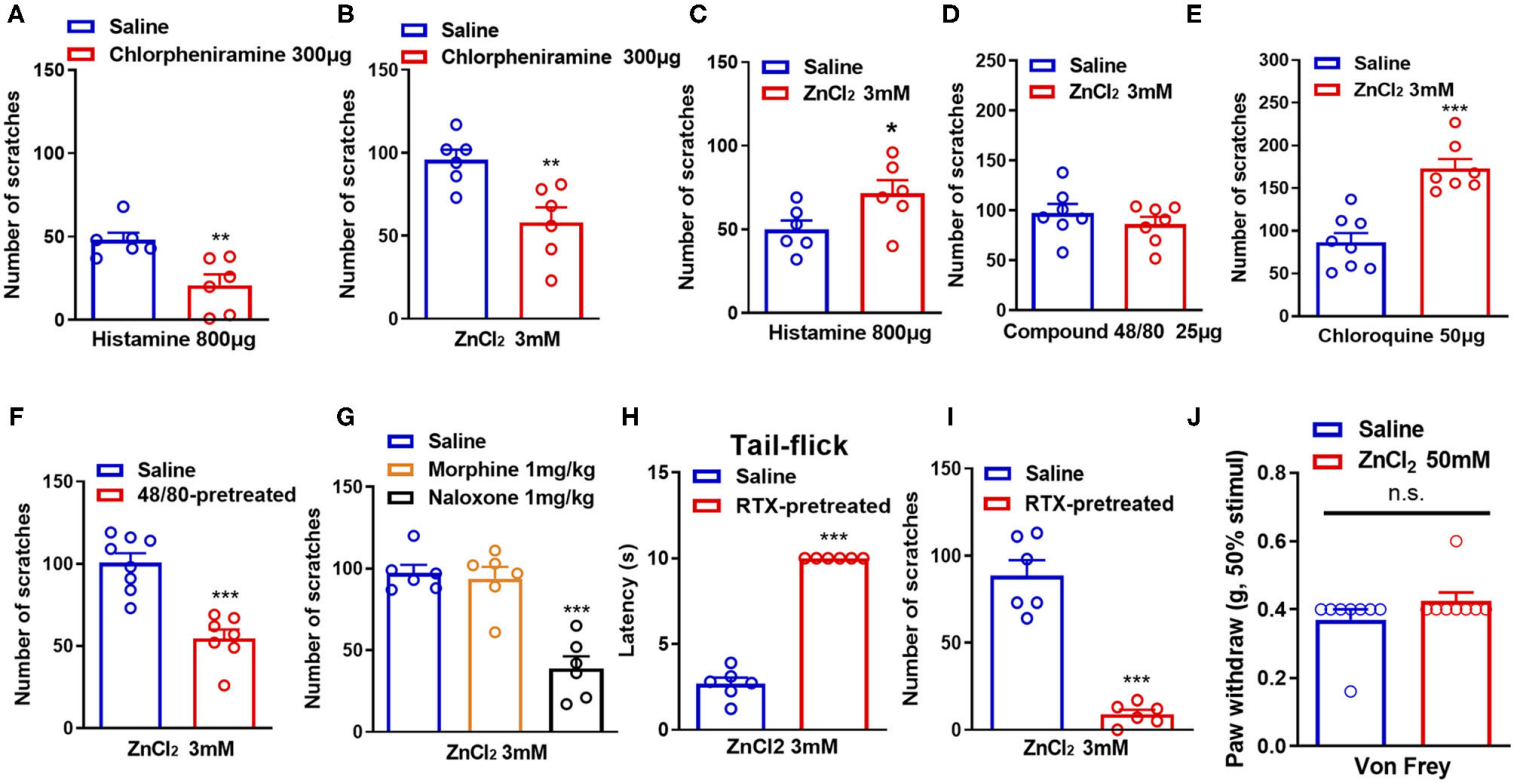
Histamine, mast cells, opioid receptors, and capsaicin-sensitive C-fibers participate in ZnCl_2_-induced itch in mice. **(A)** The effects of coadministration of a histamine H1 receptor antagonist chloropheniramine (300 μg) on histamine (800 μg)-induced itch in mice. **(B)** The effects of coadministration of chloropheniramine (300 μg) on ZnCl_2_ (3 mM)-induced itch in mice. **(C–E)** The effects of coadministration of ZnCl_2_ (3 mM) on histamine [800 μg; **(C)**]-, compound 48/80 [25 μg; **(D)**]-, chloroquine [50 μg; **(E)**]-induced itch in mice. (**F**) Pretreatment of compound 48/80 (200 μg) on ZnCl_2_ (3 mM)-induced itch in mice. **(G)** The effects of systemic administration of morphine (1 mg/kg) and naloxone (1 mg/kg) on ZnCl_2_ (3 mM)-induced itch in mice. **(H)** The effects of systemic administration of resiniferatoxin (RTX) on the latency time of tail flick response to hot water (52°C). **(I)** The effects of C-fiber depletion on ZnCl_2_ (3 mM)-induced itch in mice. **(J)** After intraplantar injection of ZnCl_2_ (50 mM, 20 μl) in the hind paw, the Von Frey test was used to detect the mechanical pain threshold in mice (*P < 0.05, **P < 0.01, ***P < 0.001 vs. Saline, unpaired Student's *t*-test; *n* = 6–9 per group). All data are expressed by means ± SEM. n.s., not significant.

Histamine has long been considered as the “gold standard” itch mediator and has been studied for more than 100 years (Dong and Dong, 2018). We then investigated whether histamine was involved in Zn^2+^-induced itch in mice. Coadministration of histamine (800 μg) and a receptor H1 antagonist chlorpheniramine (300 μg) in the nape of the neck of mice showed that chlorpheniramine attenuated histamine-induced itch in mice (*t*_10_ = 3.444, *P* = 0.0063; Figure 2A). Co-administration of ZnCl_2_ (3 mM) and chlorpheniramine (300 μg) also attenuated ZnCl_2_-induced itch in mice (*t*_10_ = 3.410, *P* = 0.0067; Figure 2B). In addition, histamine-induced itch was significantly increased by the coadministration of ZnCl_2_ (3 mM) and histamine (800 μg) in the nape of the neck of mice (*t*_10_ = 2.241, *P* = 0.0489; Figure 2C), but compound 48/80-induced itch was not affected (*t*_12_ = 0.9266, *P* = 0.3724; Figure 2D). Moreover, the coadministration of ZnCl_2_ (3 mM) and chloroquine (50 μg) in the nape of the neck of mice significantly increased the chloroquine-induced itch in mice (*t*_13_ = 5.553, *P* < 0.0001; Figure 2E).

Mast cells are important effector cells in allergic reactions and immunity, and increasing evidence supports their role in neurogenic inflammation leading to pain and itch (Meixiong et al., 2019). Mast cells contain proteases, histamine, lipids, cytokines, and chemokines, which are released by degranulation and/or non-degranulation (Gupta and Harvima, 2018). To further investigate the role of mast cells in ZnCl_2_-induced itch in mice, we depleted mast cells by the pretreatment of compound 48/80 in mice (Liu et al., 2010). The results showed that ZnCl_2_-induced itch was reduced in compound 48/80-pretreated mice (*t*_13_ = 5.621, *P* < 0.0001; Figure 2F), which suggested that mast cells may be partially involved in ZnCl_2_-induced itch in mice.

Endogenous opioids serve as neurotransmitters, hormones, and immunomodulators and can be divided into the following three classes: endorphins, dynorphins, and enkephalins (Al-Hasani and Bruchas, 2011). They bind to and activate μ-, κ-, and/or δ-opioid receptors, which are widely distributed in the central nervous system (CNS) and the peripheral nervous system (PNS) (Al-Hasani and Bruchas, 2011). Activation of opioid receptors reduces neuronal excitability through the inhibition of voltage-dependent Ca^2+^ channels and adenyl cyclase, and the activation of K^+^ channels (Al-Hasani and Bruchas, 2011). Regarding the primary afferent neurons in the skin, this reduced excitability by the activation of opioid receptors would lead to an inhibition of pain (Ikoma et al., 2006). To explore whether opioid receptors are involved in Zn^2+^-induced itch in mice, intraperitoneal (i.p) injection of μ-opioid receptors agonist morphine or the μ-opioid receptors antagonist naloxone were applied 30 min before i.d. injection of ZnCl_2_ (3 mM). The result showed that pretreatment of morphine (1 mg/kg) did not affect ZnCl_2_-induced itch (*t*_10_ = 0.4009, *P* = 0.6969; Figure 2G), while pretreatment of naloxone (1 mg/kg) significantly reduced ZnCl_2_-induced itch in mice (*t*_10_ = 6.540, *P* < 0.0001; Figure 2G).

Itch sensation is conducted from the superficial skin to the CNS mainly by unmyelinated C-fibers and some extent by small Aδ-fibers (Akiyama and Carstens, 2013). Accordingly, it is easily assumed that the damage of C-fibers may have a large impact on itch sensation (Hashimoto and Yosipovitch, 2019). To explore whether C-fibers are involved in Zn^2+^-induced itch in mice, we eliminated capsaicin-sensitive C-fibers by i.p. injection of resiniferatoxin (RTX) into mice, and the latency of tail-flick response to hot water (52°C), that was more than 10 s, confirmed the functional elimination of C-fibers (*t*_10_ = 20.04, *P* < 0.0001; Figure 2H). In addition, depletion of C-fibers by the pretreatment of RTX significantly abolished ZnCl_2_-induced itch in mice (*t*_10_ = 8.785, *P* < 0.0001; Figure 2I). Additionally, the Von Frey test showed that intraplantar injection of ZnCl_2_ (50 mM in 20 μl saline) into the hind paw did not affect the mechanical pain threshold in mice (*t*_14_ = 1.408, *P* = 0.1808; Figure 2J), which suggested that the peripheral administration of ZnCl_2_ may not be sufficient for inducing mechanical pain hypersensitivity.

### TRPA1 (but Not TRPV1 or TRPV4) Was Critically Involved in Zn^2+^-Induced Itch in Mice

**Figure 3:**
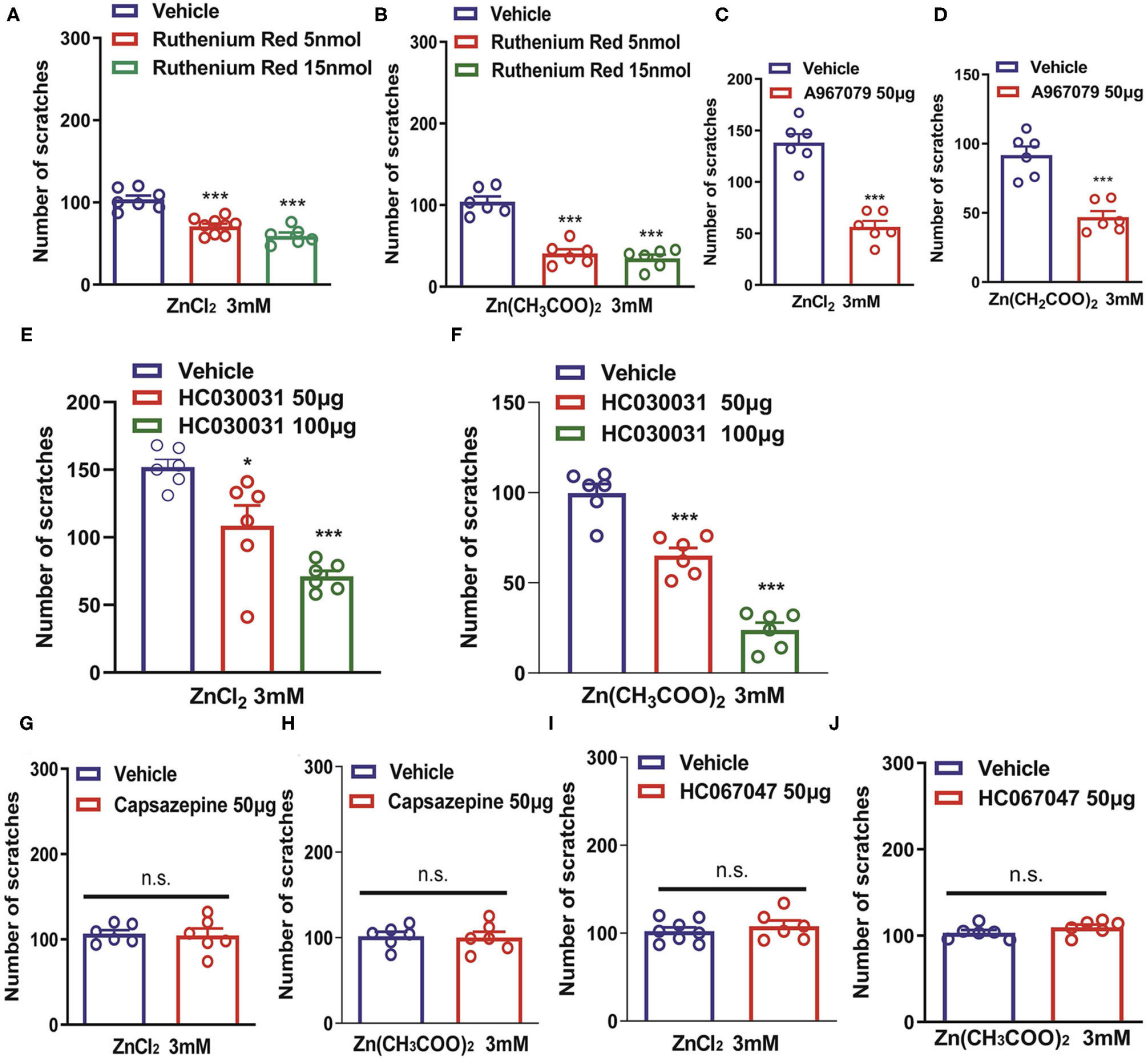
Transient receptor potential A1 (TRPA1) is required for Zn^2+^-induced itch in mice. **(A,B)** The effects of coadministration of Ruthenium Red on ZnCl_2_ (3 mM)-induced **(A)** and Zn(CH_3_COO)_2_ (3 mM)-induced itch **(B)** in mice (***P < 0.001 vs. Vehicle, one-way AVOVA following *post-hoc* Bonferroni's test; *n* = 6–9 per group). **(C,D)** The effects of coadministration of a TRPA1 blocker, A967079 on ZnCl_2_ [**(C)**; 3 mM]-induced and Zn(CH_3_COO)_2_ [**(D)**; 3 mM]-induced itch in mice (***P < 0.001 vs. Vehicle, unpaired Student's *t*-test; *n* = 6–8 per group). **(E,F)** The effects of coadministration of a TRPA1 blocker, HC030031 (50 and 100 μg) on ZnCl_2_- [**(E)**; 3 mM] and Zn(CH_3_COO)_2_- [**(F)**; 3 mM] induced itch in mice (*P < 0.05, ***P < 0.001 vs. Vehicle, one-way AVOVA following *post-hoc* Bonferroni's test; *n* = 6–9 per group). **(G,H)** The effects of coadministration of a TRPV1 blocker capsazepine on ZnCl_2_ [**(G)**; 3 mM]- and Zn(CH_3_COO)_2_ [**(H)**; 3 mM]-induced itch in mice. **(I,J)** The effects of coadministration of a TRPV4 blocker, HC067047 on ZnCl_2_ [**(I)**; 3 mM]- and Zn(CH_3_COO)_2_ [**(J)**; 3 mM]-induced itch in mice (Unpaired Student's *t*-test; *n* = 6–8 per group). All data are expressed by means ± SEM. n.s., not significant.

Transient receptor potential channels have been shown to play critical roles in various sensory functions including vision, olfaction, thermosensation, taste, mechanosensation, pain, and itch (Sun and Dong, 2016). We further investigated the roles of TRP channels in Zn^2+^-induced itch. Coadministration of a pan-TRP channel blocker, Ruthenium Red (RR; 5 and 15 nmol) and ZnCl_2_ (3 mM) into the nape of the neck of mice significantly reduced ZnCl_2_-induced itch [*F*_(2,19)_ = 30.62, *P* < 0.0001; Figure 3A]. Coadministration of Ruthenium Red (5, 15 nmol) and Zn(CH_3_COO)_2_ (3 mM) into the nape of the neck of mice also significantly reduced Zn(CH_3_COO)_2_-induced itch in mice [*F*_(2,15)_ = 47.24, *P* < 0.0001; Figure 3B]. Coadministration of a TRPA1 blocker, A967079 (50 μg) significantly reduced ZnCl_2_- and Zn(CH_3_COO)_2_-evoked acute itch in mice [For ZnCl_2_: *t*_10_ = 7.871, *P* < 0.0001; For Zn(CH_3_COO)_2_: *t*_10_ = 5.843, *P* = 0.0002; Figures 3C,D]. In addition, coadministration of another TRPA1 blocker, HC030031 (50 and 100 μg) dose-dependently inhibited ZnCl_2_- and Zn(CH_3_COO)_2_-induced itch in mice [For ZnCl_2_: *F*_(2,15)_ = 17.49, *P* = 0.0001; For Zn(CH_3_COO)_2_: *F*_(2,15)_ = 69.57 *P* < 0.0001; Figures 3E,F]. In contrast, we found that the coadministration of a TRPV1 blocker, capsazepine (50 μg) failed to affect the itching behavior evoked by ZnCl_2_ and Zn(CH_3_COO)_2_ in mice [For ZnCl_2_: *t*_10_ = 0.2132, *P* = 0.8354; For Zn(CH_3_COO)_2_:*t*_10_ = 0.1744, *P* = 0.8650; Figures 3G,H]. Similarly, coadministration of a TRPV4 blocker, HC067047 (50 μg) failed to affect the itching behavior evoked by ZnCl_2_ and Zn(CH_3_COO)_2_ in mice [For ZnCl_2_:*t*_12_ = 0.7413, *P* = 0.4728; For Zn(CH_3_COO)_2_: *t*_10_ = 1.316, *P* = 0.2176; Figures 3I,J].

**Figure 4:**
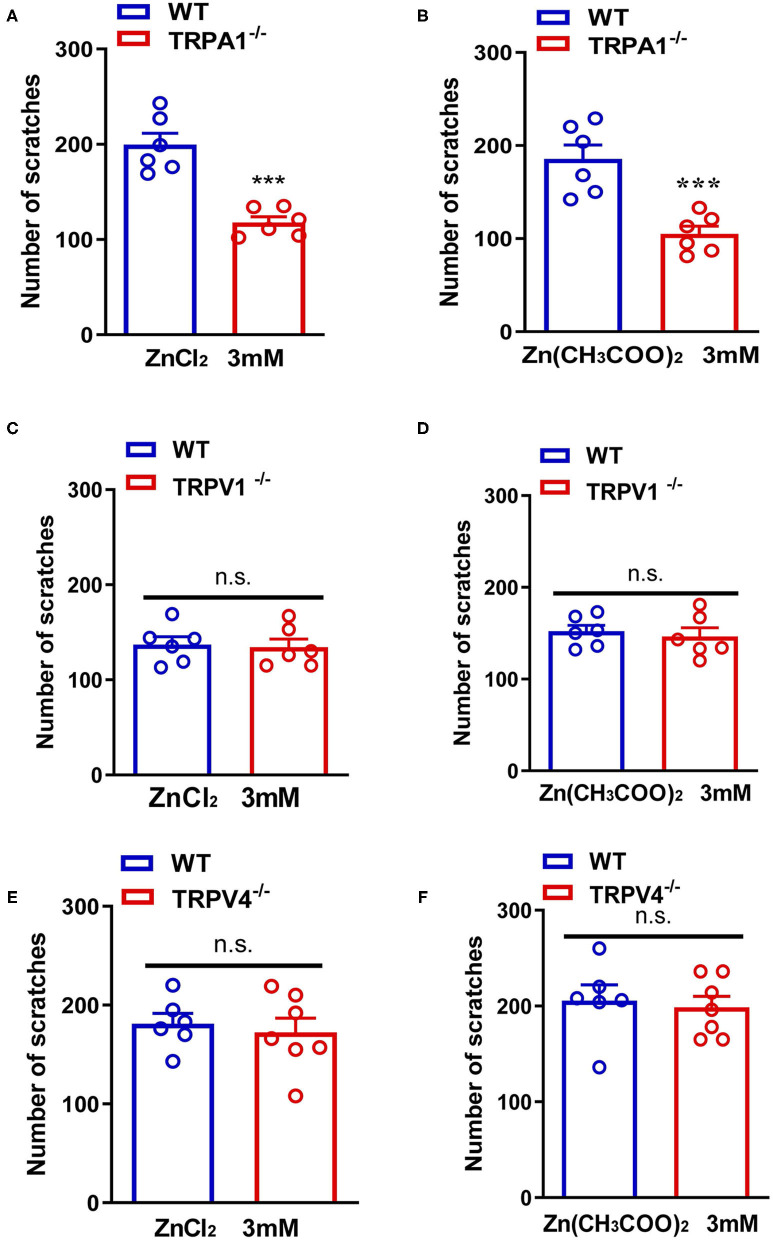
Transient receptor potential A1 (TRPA1) was involved in Zn^2+^ induced itch in mice, but TRPV1 and TRPV4 were not involved. **(A,B)** ZnCl_2_ [**(A)**; 3 mM]-evoked and Zn(CH_3_COO)_2_ [**(B)**; 3 mM]-evoked acute itch were reduced in *Trpa1*^−/−^ mice. **(C,D)** ZnCl_2_ [**(C)**; 3 mM]-evoked and Zn(CH_3_COO)_2_ [**(D)**; 3 mM]-evoked acute itching were not expressed in *Trpv1*^−/−^ mice. **(E,F)** ZnCl_2_ [**(E)**; 3 mM]-evoked and Zn(CH_3_COO)_2_ [**(F)**; 3 mM]-evoked acute itch were also not expressed in *Trpv4*^−/−^ mice (***P < 0.001 vs. WT mice, unpaired Student's *t*-test; *n* = 6–8 per group). All data are expressed by means ± SEM. n.s., not significant.

In addition, ZnCl_2_- and Zn(CH_3_COO)_2_-evoked itching behavior were abolished in *Trpa1*^−/−^ mice compared with that of wild-type (WT) mice [For ZnCl_2_: *t*_10_ = 6.054, *P* = 0.0001; For Zn(CH_3_COO)_2_: *t*_10_ = 4.651, *P* = 0.0009; Figures 4A,B]. In sharp contrast, ZnCl_2_- and Zn(CH_3_COO)_2_-evoked itching behavior had no significant difference between WT and *Trpv1*^−/−^ mice [For ZnCl_2_: *t*_10_ = 0.2377, *P* = 0.8169; For Zn(CH_3_COO)_2_: *t*_10_ = 0.4894, *P* = 0.6351; Figures 4C,D]. Similarly, ZnCl_2_- and Zn(CH_3_COO)_2_-evoked acute itching behavior were also not affected in *Trpv4*^−/−^ mice compared with that of WT mice [For ZnCl_2_: *t*_11_ = 0.4754, *P* = 0.6438; for Zn(CH_3_COO)_2_: *t*_11_ = 0.3608, *P* = 0.7251; Figures 4E,F].

### Zn^2+^ Chelators Attenuated Acute and Chronic Itch in Mice

**Figure 5:**
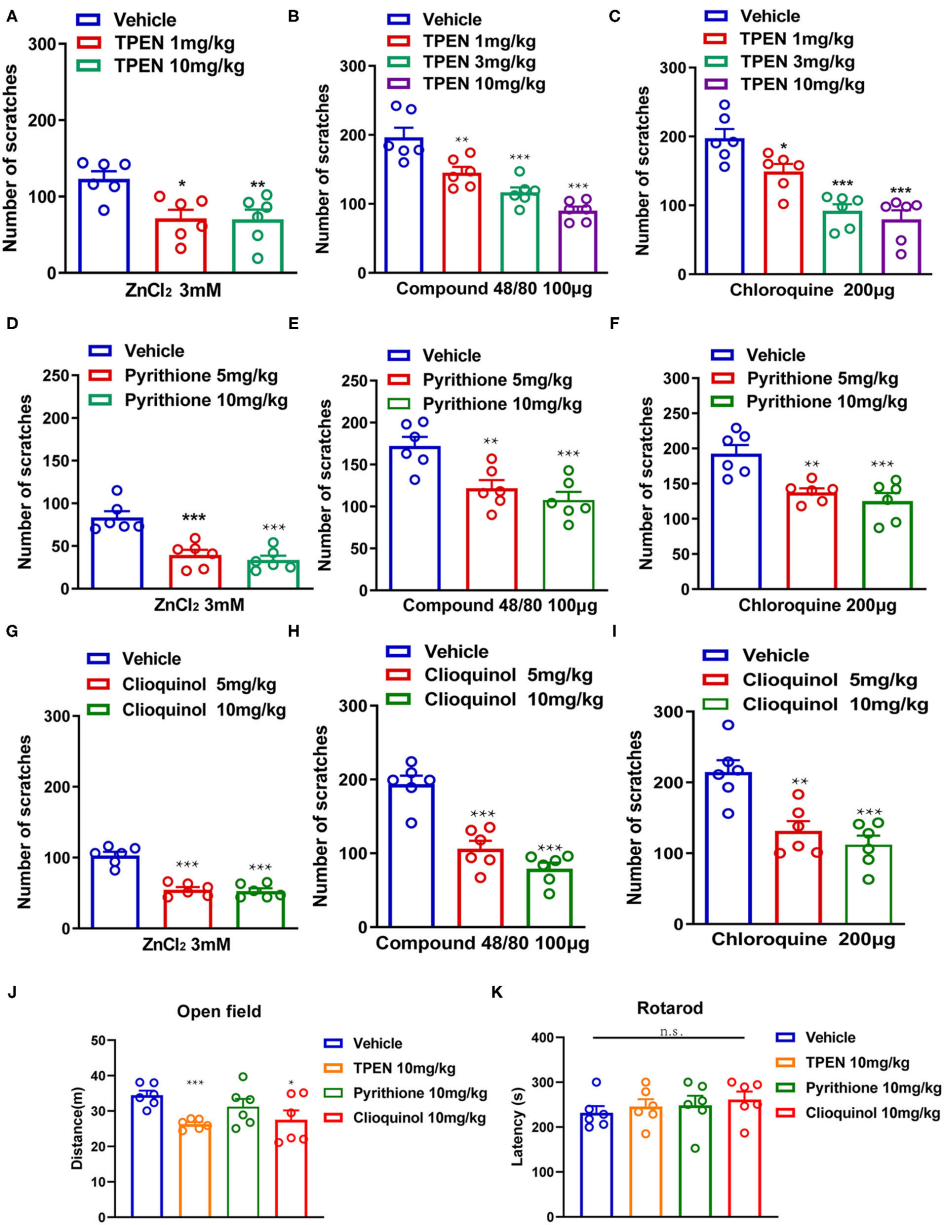
Zinc chelators attenuated acute itching behavior in mice. **(A–C)** The effects of intraperitoneal (i.p.) injection of TPEN (1–10 mg/kg) on ZnCl_2_ [**(A)**; 3 mM], compound 48/80 [**(B)**; 100 μg], and chloroquine-induced itch [**(C)**; 200 μg] in mice. **(D–F)** The effects of pyrithione (5–10 mg/kg; i.p.) on ZnCl_2_ [**(D)**; 3 mM], compound 48/80 [**(E)**; 100 μg], and chloroquine-induced itch [**(F)**; 200 μg] in mice. **(G–I)** The effects of clioquinol (5–10 mg/kg; i.p.) on ZnCl_2_ [**(G)**; 3 mM], compound 48/80 [**(H)**; 100 μg], and chloroquine-induced itch [**(I)**; 200 μg] in mice (*P < 0.05, **P < 0.01, ***P < 0.001 vs. vehicle, one-way AVOVA following *post-hoc* Bonferroni's test; *n* = 6 per group). **(J)** Open field test was performed after systemic administration of TPEN (10 mg/kg), pyrithione (10 mg/kg), and clioquinol (10 mg/kg) in mice. **(K)** Rotarod test was performed after i.p. injection of TPEN (10 mg/kg), pyrithione (10 mg/kg), and clioquinol (10 mg/kg) (*P < 0.05, ***P < 0.001 vs. Vehicle, unpaired Student's *t*-test; *n* = 6–7 per group). All data are expressed by means ± SEM. n.s., not significant.

To further investigate whether endogenous Zn^2+^ is involved in acute itch in mice, three Zn^2+^ chelators (TPEN, pyrithione, and clioquinol) were administered before i.d. injection of ZnCl_2_ (3 mM), compound 48/80 (100 μg), and chloroquine (200 μg) in mice. The result showed that TPEN (1–10 mg/kg) significantly attenuated the acute itch induced by ZnCl_2_ [*F*_(2,15)_ = 7.133, *P* = 0.0067; Figure 5A], compound 48/80 [*F*_(3,20)_ = 22.85, *P* < 0.0001; Figure 5B], and chloroquine [*F*_(3,20)_ = 20.66, *P* < 0.0001; Figure 5C] in mice. Zn^2+^ chelator pyrithione (5–10 mg/kg) also significantly attenuated the acute itch induced by ZnCl_2_ [*F*_(2,15)_ = 19.79, *P* < 0.0001; Figure 5D], compound 48/80 [*F*_(2,15)_ = 11.13, *P* = 0.0011; Figure 5E], and chloroquine [*F*_(2,15)_ = 12.24, *P* = 0.0007; Figure 5F] in mice. Moreover, clioquinol (5–10 mg/kg) significantly reduced the itch evoked by ZnCl_2_ [*F*_(2, 15)_ = 43.70, *P* < 0.0001; Figure 5G], compound 48/80 [*F*_(2,15)_ = 33.69, *P* < 0.0001; Figure 5H], and chloroquine [*F*_(2,15)_ = 13.89, *P* = 0.0004; Figure 5I] in mice.

We tested the potential side effects of systemic administration of Zn^2+^ chelators in mice by using open field test and Rotarod test. For open field test, systemic administration of pyrithione (10 mg/kg) showed no effect on the locomotion of mice, but TPEN (10 mg/kg) and clioquinol (10 mg/kg) showed slight but significant inhibition on the locomotion of mice [For TPEN: *t*_10_ = 5.802, *P* = 0.0002; for pyrithione: *t*_10_ = 1.274, *P* = 0.2314; For clioquinol: *t*_10_ =2.356, *P* = 0.0402; Figure 5J]. The Rotarod test showed that systemic administration of all tested Zn^2+^ chelators had no effect on the motor function of mice ([For TPEN: *t*_12_ = 0.6283, *P* = 0.5416; for pyrithione: *t*_12_ = 0.4426, *P* = 0.6659; For clioquinol: *t*_12_ =1.996, *P* = 0.0691; Figure 5K]. Thus, the results indicated that a systemic administration of Zn^2+^ chelators may produce limited side effects in mice.

**Figure 6:**
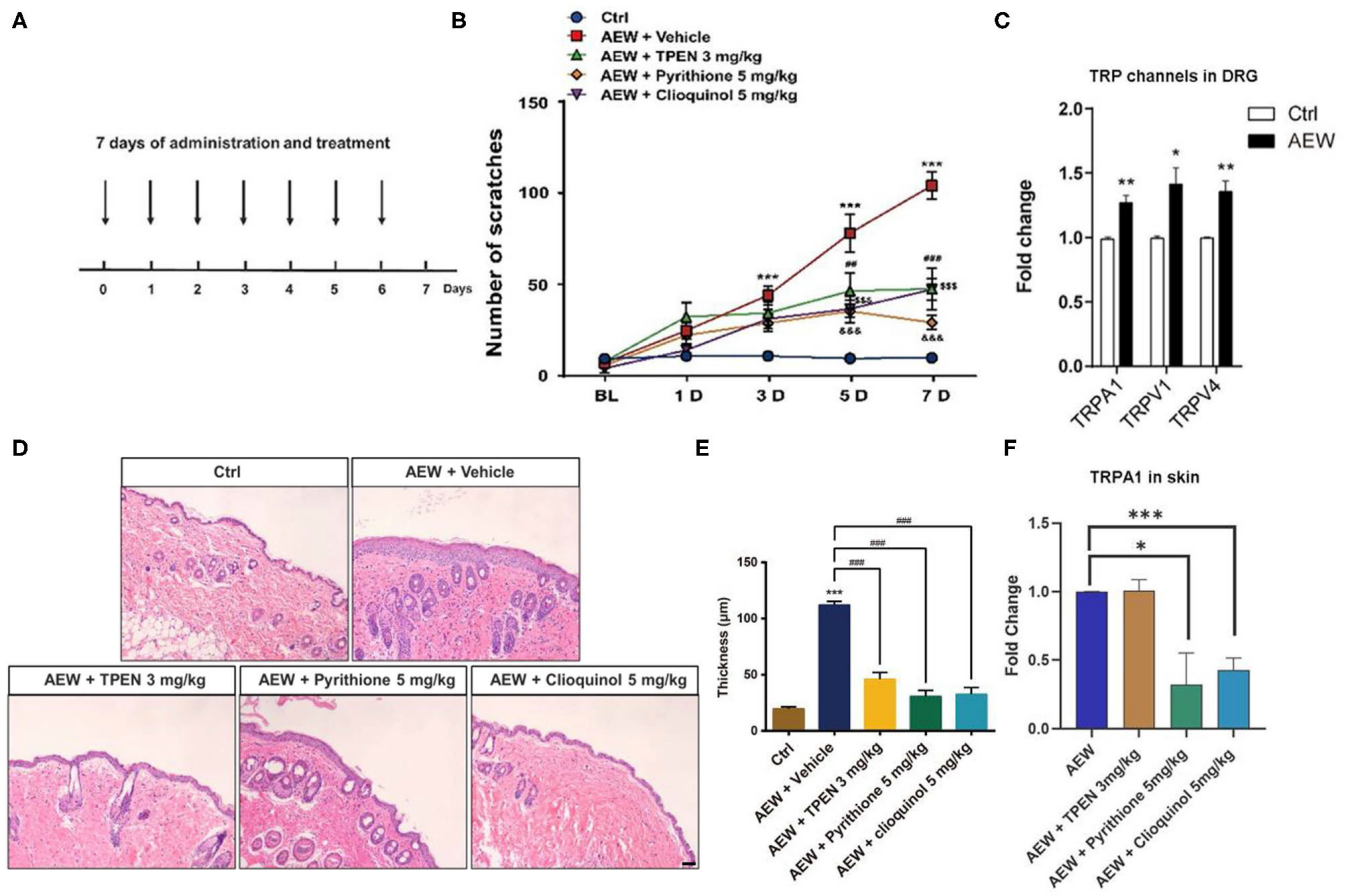
The effects of the administration of three Zn^2+^ chelating agents on dry skin chronic model. **(A)** The establishment of dry skin-induced chronic itch model. **(B)** The three Zn^2+^ chelators, such as TPEN (3 mg/kg), pyrithione (5 mg/kg), and clioquinol (5 mg/kg) can significantly inhibit the chronic itching behavioral response of mice induced by dry skin (***P < 0.001 vs. Ctrl, ^##^*P* < 0.01, ^###^*P* < 0.001, ^$$$^ *P* < 0.001, ^&&&^*P* < 0.001 vs. AEW + Vehicle, two-way ANOVA following *post-hoc* Bonferroni's test; *n* = 6–10 per group). **(C)** The mRNA expression levels of *Trpa1, Trpv1*, and *Trpv4* in the DRG of dry skin induced chronic itch model (*P < 0.05, **P < 0.01 vs. Ctrl, unpaired Student's *t*-test; *n* = 5 per group). **(D)** The effects of treatment with Zn^2+^ chelators on the epidermal thickness of the dry skin induced chronic itch model. (*P < 0.05, **P < 0.01, ***P < 0.001 vs. Ctrl, unpaired Student's *t-*test; *n* = 5 per group). **(E)** The epidermal thickness statistics of the dry skin model group and the treatment group (***P < 0.001 vs. Ctrl, ^###^*P* < 0.001 vs. AEW + Vehicle, unpaired Student's *t*-test; *n* = 4 per group). **(F)** Zn^2+^ chelators pyrithione (5 mg/kg) and clioquinol (5 mg/kg) can decrease *Trpa1* mRNA level, but not for TPEN (3 mg/kg) (*P < 0.05, ***P < 0.001 vs. AEW, unpaired Student's *t*-test; *n* = 5 per group). All data are expressed by means ± SEM. Ctrl, control; n.s., not significant.

In addition, we employed a dry skin-induced chronic itch model to explore whether endogenous Zn^2+^ is involved in chronic itch in mice by daily systemic administration of three Zn^2+^ chelators in mice, such as TPEN (3 mg/kg), pyrithione (5 mg/kg), and clioquinol (5 mg/kg) (Figure 6A). Behavioral analysis showed that all Zn^2+^ chelators significantly reduced the chronic itch induced by dry skin in mice [Time: *F*_(4,160)_ = 46.59, *P* < 0.0001; Treatment: *F*_(4,160)_ = 53.17, *P* < 0.0001; Interaction: *F*_(16,160)_ = 10.40, *P* < 0.0001; Figure 6B]. Our results revealed that the mRNA expression levels of *Trpa1* (*t*_8_ = 4.798, *P* = 0.0014), *Trpv1* (*t*_8_ = 3.258, *P* = 0.0116), and *Trpv4* (*t*_8_ = 4.297, *P* = 0.0026) significantly increased in the DRGs of dry skin-induced chronic itch model (Figure 6C).

Moreover, H&E staining showed that all three Zn^2+^ chelators, such as TPEN (3 mg/kg), pyrithione (5 mg/kg), and clioquinol (5 mg/kg) significantly alleviated the increased epidermal thickness induced by AEW treatment in mice (*t*_4_ = 9.664, *P* = 0.0006; *t*_4_ = 12.91, *P* = 0.0002; *t*_4_ = 11.43, *P* = 0.0003; Figures 6D,E). We also found that the mRNA expression level of *Trpa1* in the skin of AEW-treated mice was significantly inhibited by pyrithione (5 mg/kg) and clioquinol (5 mg/kg), but not for TPEN (3 mg/kg) (For TPEN: *t*_8_ = 0.09126, *P* = 0.9295; for pyrithione: *t*_8_ = 2.941, *P* = 0.0187; For clioquinol: *t*_8_ = 6.355, *P* = 0.0002; Figure 6F).

### The Changes of the Expression Levels of ZIPs, ZnTs, and TRP Channels in a Dry Skin-Induced Chronic Itch Mouse Model

Zinc is one of the most important trace elements in the organism and usually exists in cells as Zn^2+^. Zinc homeostasis in mammals is primarily maintained through Zn^2+^ transporters that are reasonable for regulating cellular uptake, efflux, and intracellular trafficking of Zn^2+^ (Kambe et al., 2015). There are two major Zn^2+^ transporter/carrier families known as *Slc30* and *Slc39* (Kambe et al., 2015). The ZIPs are encoded by the *Slc39a* family, while ZnTs are encoded by the *Slc30a* family. The ZnTs family, comprised of 10 members in humans, is involved in transporting zinc from the cytosol to the extracellular space or into intracellular organelles. The ZIPs family, comprised of 14 members, is primarily involved in transporting Zn^2+^ from the extracellular space or intracellular organelles into the cytosol and therefore increases the cytosolic zinc concentrations (Sapkota and Knoell, 2018).

**Figure 7:**
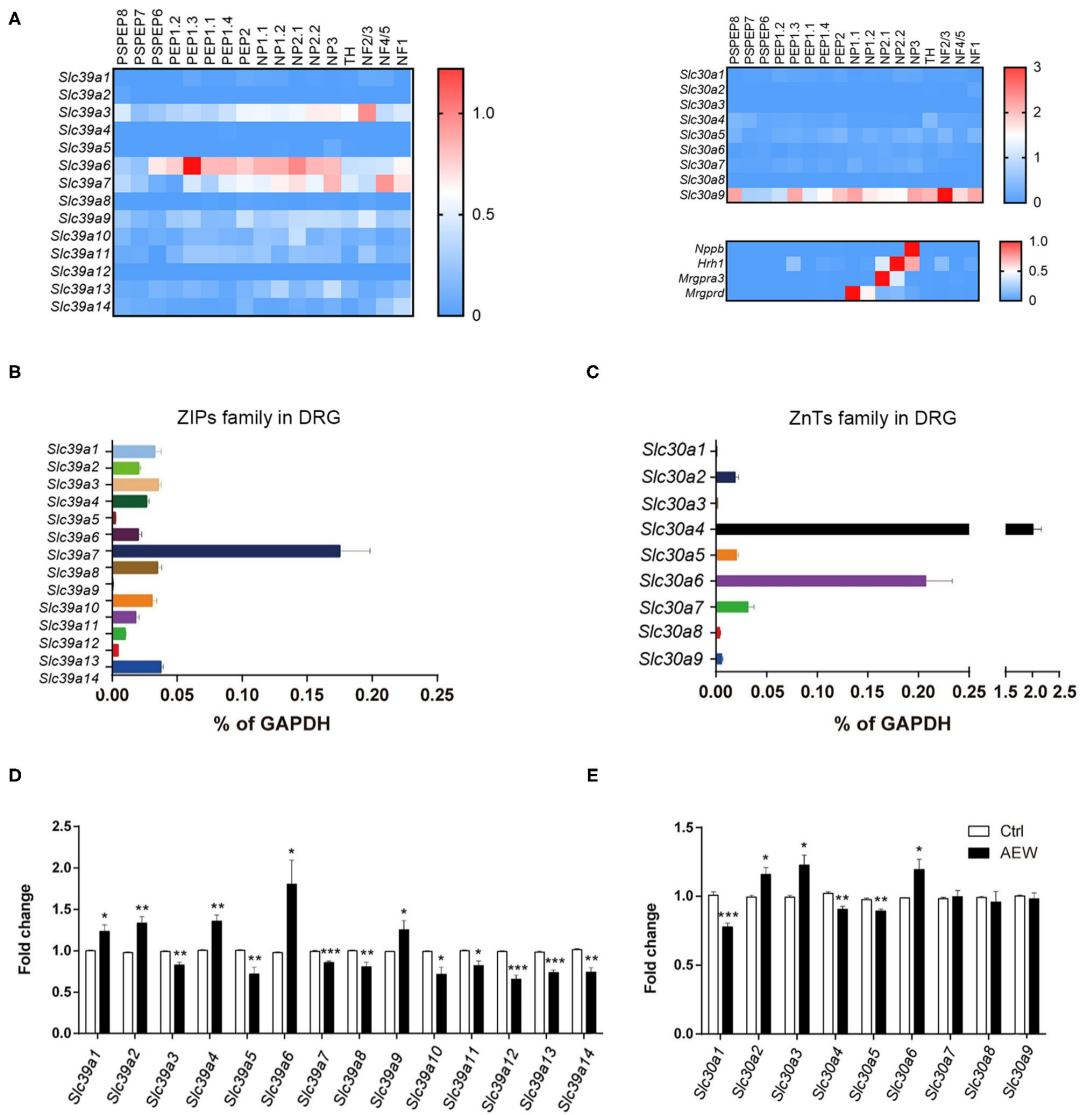
The expression of ZIPs/*Slc39as* and ZnTs/*Slc30as* in the DRG of dry skin induced chronic itch model. **(A)** The gene expression of ZIPs and ZnTs family and itch marker in the DRG neuron of mouse based on previous published single cell RNA-seq database. **(B,C)** The mRNA expression levels of **(B)** *Slc39as* and **(C)** *Slc30as* in the DRG of WT mice. **(D,E)** The mRNA expression levels of **(D)** *Slc39as* and **(E)** *Slc30as* in the DRG of dry skin induced chronic itch model (*P < 0.05, **P < 0.01, ***P < 0.001 vs. Ctrl, unpaired Student's *t*-test; *n* = 5 per group). All data are expressed by means ± SEM. Ctrl, control; n.s., not significant.

Based on the single-cell RNA sequencing (RNA-seq) data from a previous study (Zeisel et al., 2018), it was found that ZIPs and ZnTs family were differently expressed in the DRG neuron of the mouse (Figure 7A). We detected the mRNA expression levels of *Slc39a* family and *Slc30a* family of DRG in wild-type (WT) mice. The results showed that *Slc39a7* had the relative highest expression level in *Slc39a* family (Figure 7B), and *Slc30a4* and *Slc30a6* had the relative highest expression levels in *Slc30a* family (Figure 7C). In addition, Q-PCR analysis showed that the mRNA expression levels of the subtypes of *Slc39a* family changed in the DRGs of dry skin-induced chronic itch model. The mRNA expression levels of *Slc*39*a*1(*t*_8_ = 2.947, *P* = 0.0185), *Slc39a2* (*t*_8_ = 4.494, *P* = 0.0020), *Slc39a4* (*t*_8_ = 4.677, *P* = 0.0016), *Slc39a6* (*t*_8_ = 2.807, *P* = 0.0230), and *Slc39a9* (*t*_8_ = 2.317, *P* = 0.0491) were significantly upregulated compared to the control group (Figure 7D). The mRNA expression levels of *Slc39a3* (*t*_8_ = 4.494, *P* = 0.0020), *Slc39a5* (*t*_8_ = 3.499, *P* = 0.0081), *Slc39a7* (*t*_8_ = 6.393, *P* = 0.0002), *Slc39a8* (*t*_8_ = 3.502, *P* = 0.0081), *Slc39a10* (*t*_8_ = 3.273, *P* = 0.0113), *Slc39a11* (*t*_8_ = 3.219, *P* = 0.0123), *Slc39a12* (*t*_8_ = 6.940, *P* = 0.0001), *Slc39a13* (*t*_8_ = 7.493, *P* < 0.0001), and *Slc39a14* (*t*_8_ = 4.677, *P* = 0.0016) were significantly downregulated (Figure 7D). The Q-PCR results showed that the mRNA expression of the subtypes of *Slc30as* changed in the DRGs of dry skin-induced chronic itch model. The mRNA expression levels of *Slc30a1* (*t*_8_ = 6.096, *P* = 0.0003), *Slc30a4* (*t*_8_ = 4.638, *P* = 0.0017), and *Slc30a5* (*t*_8_ = 4.270, *P* = 0.0027) were significantly decreased compared with the control group (Figure 7E). However, the mRNA expression levels of *Slc30a2* (*t*_8_ = 3.215, *P* = 0.0123), *Slc30a3* (*t*_8_ = 3.038, *P* = 0.0161), and *Slc30a6* (*t*_8_ = 2.743, *P* = 0.0253) were significantly increased compared to the control group (Figure 7E).

### Activation of p-ERK Signaling in the DRGs and the Skin Contributed to Zn^2+^-Induced Acute and Chronic Itch in Mice

**Figure 8:**
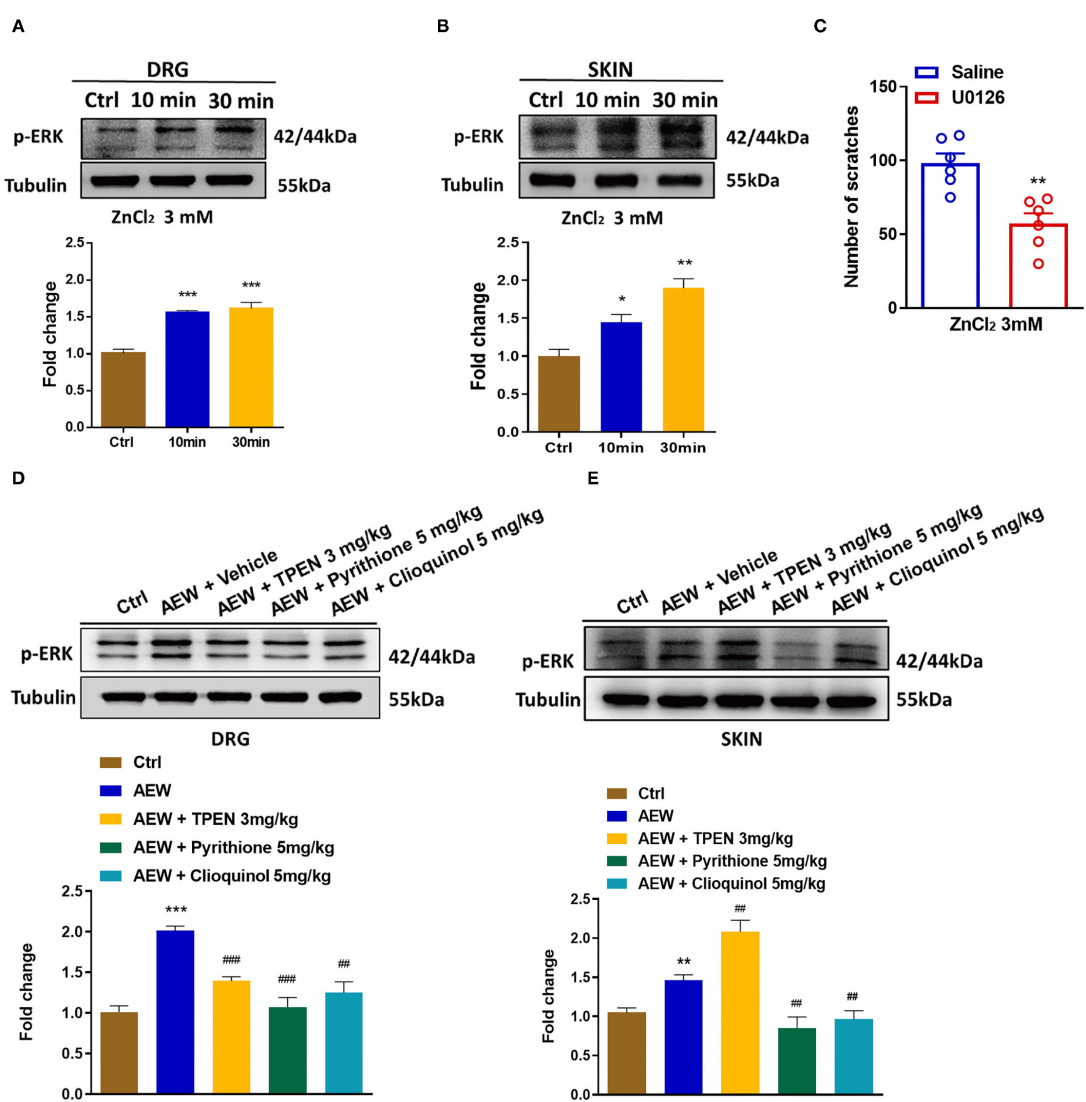
Activation of extracellular signal-regulated kinase (ERK) signaling was involved in Zn^2+^-mediated acute and chronic itch in mice. **(A,B)** Western blots (upper panel) and quantification (lower panel) shows that p-ERK expression is significantly increased at 10 and 30 min in DRG **(A)** and skin **(B)** after i.d. injection of ZnCl_2_(3 mM) (*P < 0.05, **P < 0.01, ***P < 0.001 vs. Ctrl, unpaired Student's *t*-test; *n* = 4). **(C)** Intrathecal injection of U0126 (1 nmol) decreased the ZnCl_2_-induced scratching behavior in mice by 3 mM (**P < 0.01 vs. Saline, unpaired Student's *t*-test; *n* = 6). **(D,E)** Western blots (upper panel) and quantification (lower panel) show that the expression of p-ERK in DRG **(D)** and skin **(E)** of dry skin induces chronic itch model (**P < 0.01, ***P < 0.001 vs. Ctrl, ^##^*P* < 0.01, ^###^*P* < 0.001 vs. AEW + Vehicle, unpaired Student's *t*-test; *n* = 4 per group). All data are expressed by means ± SEM. Ctrl, control; n.s. not significant.

Previous reports have shown that phosphorylation of ERK in the DRGs and the spinal cord contributes to the genesis of itch (Zhang et al., 2014) and pain (Wang et al., 2018). In the current study, Western blotting analysis showed that i.d. injection of ZnCl_2_ (3 mM) upregulated the expression of p-ERK in both the DRG [For 10 min: *t*_6_ = 9.007, *P* = 0.0001; For 30 min: *t*_6_ = 6.185, *P* = 0.0008; Figure 8A] and the skin (For 10 min: *t*_6_ = 3.232, *P* = 0.0179; For 30 min: *t*_6_ = 5.898, *P* = 0.0011; Figure 8B). Moreover, intrathecal (i.t.) injection of the mitogen-activated protein kinase (MEK) inhibitor, U0126 (1 nmol) inhibited ZnCl_2_-induced acute itch in mice (*t*_10_ = 4.237, *P* = 0.0017; Figure 8C). In addition, the expression levels of p-ERK were upregulated in the DRGs (*t*_6_ = 10.54, *P* < 0.0001; Figure 8D) and the skin (*t*_6_ = 4.488, *P* = 0.0042; Figure 8E) of dry skin mice. Administration of three Zn^2+^ chelators, such as TPEN (3 mg/kg), pyrithione (5 mg/kg), and clioquinol (5 mg/kg) significantly downregulated the p-ERK in the DRGs of dry skin mice (*t*_6_ = 8.165, *P* = 0.0002; *t*_6_ = 5.272, *P* = 0.0019; *t*_6_ = 7.286, *P* = 0.0003; Figure 8D). In addition, pyrithione (5 mg/kg) and clioquinol (5 mg/kg) significantly reduced the expression levels of p-ERK in the skin of dry skin-induced chronic itch model (*t*_6_ = 3.852, *P* = 0.0084; *t*_6_ = 3.872, *P* = 0.0082; Figure 8E). In contrast, TPEN (3 mg/kg) upregulated the expression levels of p-ERK in the skin of dry skin-induced chronic itch model (*t*_6_ = 3.839, *P* = 0.0086; Figure 8E).

### GPR39 Was Involved in Dry Skin-Induced Chronic Itch in Mice

**Figure 9:**
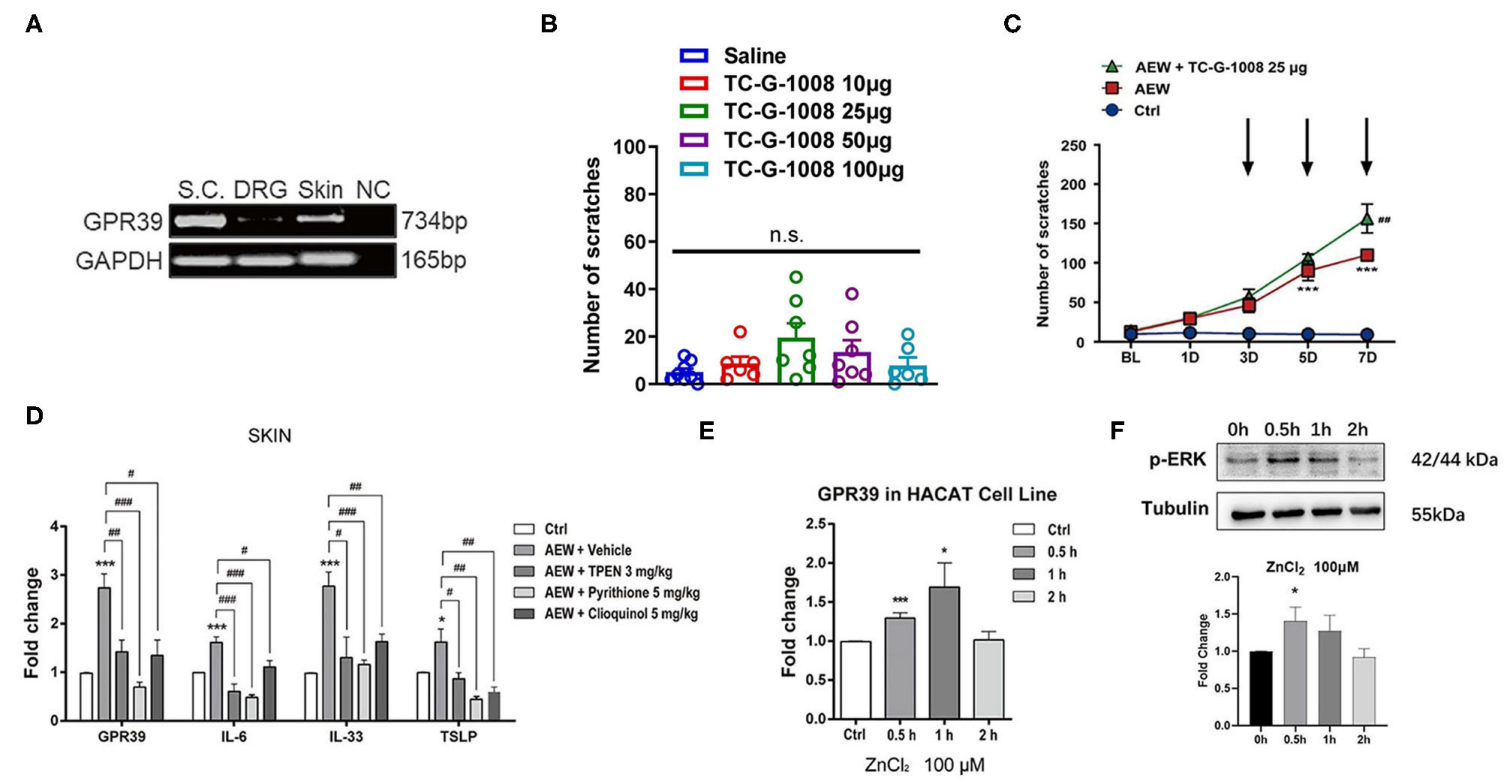
GPR39 was possibly involved in dry skin-induced chronic itch in mice. **(A)** The RT**-**PCR results showed that GPR39 is expressed in the spinal cord and the skin of mice, and only little in the DRG. **(B)** A GPR39 agonist, TC-G-1008 (1–100 μg) was not able to evoke acute itch in mice (one-way AVOVA following *post-hoc* Bonferroni's test; *n* = 6–8). **(C)** TC-G-1008 (25 μg) significantly increased AEW-induced chronic itch in mice (***P < 0.001 vs Ctrl, ^##^*P* < 0.01 vs. AEW, two-way ANOVA following *post-hoc* Bonferroni's test; *n* = 8). **(D)** The expression of *Gpr39, Il-6, Il-33*, and *Tslp* in the skin of dry skin induced chronic itch model (*P < 0.05, ***P < 0.001 vs. Ctrl, ^#^*P* < 0.05, ^##^*P* < 0.01, ^###^*P* < 0.001 vs. AEW + Vehicle, unpaired Student's *t*-test; *n* = 5 per group). **(E)** In HaCaT cells, the mRNA expression levels of *Gpr39* were significantly upregulated after ZnCl_2_ (100 μM) incubated for 0.5 and 1 h. **(F)** In HaCaT cells, the protein expression levels of p-ERK were significantly upregulated after ZnCl_2_ (100 μM) incubated for 0.5 h (*P < 0.05, ***P < 0.001 vs. Ctrl, one-way AVOVA following *post-hoc* Bonferroni's test; *n* = 5–6 per group). All data are expressed by means ± SEM. Ctrl, control; n.s. not significant.

As a distinct GPCR that senses extracellular Zn^2+^, the GPR39 was shown to regulate the activity of ion transport, which is essential for the physiological function of the epithelial and neuronal cells (Hershfinkel, 2018). We further investigated whether the zinc-sensing receptor, GPR39 was involved in acute or chronic itch in mice. A reverse transcription polymerase chain reaction (RT-PCR) analysis showed that GPR39 was expressed in the spinal cord and skin of mice, with a relatively high level, but very low levels in the DRGs (Figure 9A). In order to explore the potential role of GPR39 and acute itch, the mice were i.d. injected into the nape of the neck with a selective GPR39 agonist, TC-G-1008. We found that i.d. injection of TC-G-1008 (10–100 μg) in the nape of the neck of mice was not able to evoke scratching behavior in mice [*F*_(4,29)_ = 2.043, *P* = 0.1145; Figure 9B]. As described previously, dry skin mice were induced by AEW (1:1 mixture of acetone and ether) treatment every day for seven days, and TC-G-1008 (25 μg) was i.d. injected into the neck on the 3rd, 5th, and 7th days, respectively. The behavioral analysis showed that TC-G-1008 significantly increased the itching behaviors under dry skin-induced chronic itch condition in mice [Time: *F*_(4,105)_ = 58.55, *P* < 0.0001; Treatment: *F*_(2,105)_ = 96.31, *P* < 0.0001; Interaction: *F*_(8,105)_ = 16.69, *P* < 0.0001; Figure 9C]. In addition, our study showed that the mRNA expression level of GPR39, IL-6, IL-33, and thymic stromal lymphopoietin (TSLP) in the skin of dry skin mouse model were significantly upregulated compared with that of the control group (*t*_8_ = 6.055, *P* = 0.0003; *t*_8_ = 5.267, *P* = 0.0008; *t*_8_ = 6.182, *P* = 0.0003; *t*_8_ = 2.317, *P* = 0.0492; Figure 9D). Administration of Zn^2+^ chelators, such as TPEN (3 mg/kg), pyrithione (5 mg/kg), and clioquinol (5 mg/kg) significantly downregulated the mRNA expression of GPR39 (For TPEN: *t*_8_ = 3.495, *P* = 0.0081; For pyrithione; *t*_8_ = 6.611, *P* = 0.0002; For clioquinol; *t*_8_ = 3.243, *P* = 0.0118; Figure 9D), IL-6 (For TPEN: *t*_8_ = 5.277, *P* = 0.0007; For pyrithione: *t*_8_ = 8.837, *P* < 0.0001; For clioquinol: *t*_8_=2.859, *P* = 0.0212; Figure 9D), IL-33 (For TPEN: *t*_8_ = 2.892, *P* = 0.0201; For pyrithione: *t*_8_ = 5.312, *P* = 0.0007; For clioquinol: *t*_8_ = 3.475, *P* = 0.0084; Figure 9D), and TSLP (For TPEN: *t*_8_ = 2.517, *P* = 0.0360; For pyrithione: *t*_8_ = 4.246, *P* = 0.0028; For clioquinol: *t*_8_ = 3.547, *P* = 0.0075; Figure 9D) in the skin of dry skin mouse model.

The HaCaT cell lines are immortalized human epidermal cells, which have similar differentiation characteristics to human keratinocytes (Boukamp et al., 1988). In HaCaT cells, ZnCl_2_ (100 μM) was incubated with HaCaT cells for 0.5, 1, and 2 h, and the mRNA expression levels of GPR39 was analyzed by Q-PCR. The results showed that compared to that of the control group, the mRNA expression of GPR39 was significantly upregulated by the treatment of ZnCl_2_ (100 μM) for 0.5 and 1 h (For 0.5 h: *t*_9_ = 5.055, *P* = 0.0007; For 1 h: *t*_9_ = 2.516, *P* = 0.0330; For 2 h: *t*_10_ = 0.1994, *P* = 0.8459; Figure 9E). The protein level of p-ERK was analyzed by Western blotting analysis. Compared with that of the control group, p-ERK expression in HaCaT cells was significantly upregulated by the treatment of ZnCl_2_ (100 μM) for 0.5 h (For 0.5 h: *t*_4_ = 3.718, *P* = 0.0205; For 1 h: *t*_4_ = 2.271, *P* = 0.0857; For 2 h: *t*_4_ = 1.220, *P* = 0.2895; Figure 9F).

## Discussion

As one of the most important trace metal elements in organisms, zinc plays a key role in many physiological and biochemical processes (Hinman et al., 2006; Patapoutian et al., 2009). Recent studies have reported that both exogenous and endogenous Zn^2+^ can regulate pain sensation (Luo et al., 2018). However, whether and how Zn^2+^ regulates itch signaling is rarely reported. In this study, we aimed to explore the role of Zn^2+^ in the regulation of acute and chronic itch. We found that both exogenous and endogenous Zn^2+^ were critically involved in the pathogenesis of acute and chronic itch in mice. Furthermore, we revealed that TRPA1, GPR39, and ERK signaling pathways were critically involved in Zn^2+^-mediated acute and chronic itch in mice. Thus, targeting this Zn^2+^/TRPA1/GPR39/ERK signaling pathway may be a novel strategy for anti-itch therapy.

### The Effects of Exogenous Zn^2+^ in Acute Itch

To investigate the effects of the exposure of exogenous Zn^2+^ on acute itch, we employed three different zinc compounds, such as ZnCl_2_, Zn(CH_3_COO)_2_, and ZnSO_4_. We observed the Zn^2+^-induced dose-dependent scratching behavior in mice. We further used the cheek model (Shimada and LaMotte, 2008) to define whether Zn^2+^ induced pain or itch sensation in mice. The results clearly showed that i.d. injection of Zn^2+^ into the cheek only induced itch-indicative scratching, but not pain-indicative wiping in mice, indicating that an exposure to Zn^2+^ induced pure itch sensation in the mice. Given the dosage of zinc used in our study was relatively high, our results indicated that the exposure of overdose of Zn^2+^ in the skin may induce itch sensation, which may be a skin manifestation of zinc toxicity.

### The Roles of Endogenous Zn^2+^ in Acute and Chronic Itch

So far, it is largely unknown about the roles of endogenous Zn^2+^ in the regulation of itch. Previous studies found that zinc served as a critical nutrient and played an important role in maintaining skin integrity. Clinical observation demonstrated that zinc supplementation or zinc ointments can be effective in itch relief (Chasapis et al., 2012; Roohani et al., 2013). In addition, a previous clinical study showed that serum zinc level was significantly lower in the patients with itching compared to those without itching or the control group (Takai et al., 2017). Thus, we speculated that zinc deficiency might be associated with itch, possibly due to dry skin or skin abnormality. Additionally, zinc supplementation may correct zinc deficiency in the skin and lead to itch relief. However, whether and how zinc deficiency causes itch remains unclear and warrant further investigation. In the present study, to clarify the role of endogenous Zn^2+^ in acute and chronic itch by using three different Zn^2+^ chelating agents, including TPEN, pyrithione, and clioquinol, our results demonstrated that systemic administration of Zn^2+^ chelators significantly reduced the scratching behavior induced by ZnCl_2_, compound 48/80, and chloroquine.

To investigate the role of endogenous Zn^2+^ in chronic itch, we used Zn^2+^ chelating agents to explore their effects on the dry skin-induced chronic itch model. Consistent with our current results in the acute itch model, Zn^2+^ chelators were also effective to attenuate dry skin-induced chronic itch in mice. We speculated that under pathological itch conditions, endogenous Zn^2+^ may release from skin cells (e.g., mast cells or keratinocytes) and then stimulate free nerve terminals in the skin to cause itching. Thus, endogenously released Zn^2+^ may be a significant contributor to the pathogenesis of chronic itch. It was noticed that itch could be caused by either zinc deficiency or excess zinc release, which clearly had distinct mechanisms.

Moreover, systemic administration of Zn^2+^ chelators did not affect the motor function in mice by using the Rotarod test, although some Zn^2+^ chelators (e.g., TPEN and clioquinol) slightly reduced locomotion by using open field test. The data suggested that Zn^2+^ chelators might have limited side effects. Although the cell types that release endogenous Zn^2+^ remain unclear, the Zn^2+^ chelators may be potentially useful for the clinical management of itch and warrant further investigation.

To explore the mechanisms underlying the dysregulation of Zn^2+^ homeostasis in the chronic itch model, we further investigated the expression changes of the zinc transporters family under dry skin-induced chronic itch condition. Previously, the single-cell RNA-seq data showed that several members of the ZIPs and ZnTs family were expressed by neurons in the DRGs. In our study, we found that several members of the ZIPs and ZnTs family were significantly upregulated in the DRGs under dry skin-induced chronic itch condition, including *Slc39a1, Slc39a2, Slc39a4, Slc39a6, Slc39a9, Slc30a2, Slc30a3*, and *Slc30a6*. In contrast, several members of ZIPs and ZnTs family were significantly downregulated in the DRGs under dry skin-induced chronic itch condition, including *Slc39a3, Slc39a5, Slc39a7, Slc39a8, Slc39a10, Slc39a11, Slc39a12, Slc39a13, Slc39a14, Slc30a1, Slc30a4*, and *Slc30a5*. We predicted that the changes in the expression of ZIPs and ZnTs in the DRG under chronic itch conditions may lead to zinc dyshomeostasis in the PNS, possibly contributing to the pathogenesis of chronic itch. Because we used the whole DRG tissue containing glial cells and immune cells, there is some difference in the expression levels of ZIPs (e.g., *Slc39a6* and *Slc39a7*) and ZnTs (e.g., Slc30a4, Slc30a6, and Slc30a9) between single-cell RNA-seq data and our q-PCR analysis. The precise role of certain members of the ZIP or ZnT family in itch remains unclear and warrants further investigation.

### TRPA1 Is Critically Involved in Zn^2+^-Mediated Itch in Mice

Given multiple TRP channels are expressed by capsaicin-sensitive C-fibers, we subsequently combined the pharmacological method and knockout mice to determine which TRP channel mediates Zn^2+^-induced scratching behavior in the mice. Our results showed that TRPA1 was critically involved in itching behavior induced by Zn^2+^ in mice, but not for TRPV1 and TRPV4. Previous studies showed that Zn^2+^ (EC50 ≈ 2 μM) could directly activate the TRPA1 channel (Hu et al., 2009), promoted Ca^2+^ influx, which activated primary sensory neurons, and intraplantar injection of Zn^2+^ (30 mM) induced nociceptive behaviors in the mice (Hu et al., 2009). It was noticed that the concentration of Zn^2+^ used in *in vivo* experiments was similar to our study and also much higher than that of the *in vitro* experiments. Previous studies showed that Zn^2+^-induced TRPA1 activation may not be required in the cellular toxicity of zinc *in vitro* (Steinritz et al., 2018). However, our data demonstrated that TRPA1 activation is required for Zn^2+^-induced itch in mice. Andersson et al. (2009) found that TRPA1 was also activated by clioquinol and pyrithione by increasing the intracellular Zn^2+^ in DRG sensory neurons. However, our preliminary data showed that i.d. injection of clioquinol or pyrithione did not induce scratching in mice (data not shown). Thus, there are some inconsistencies between *in vivo* and *in vitro* studies. Moreover, our results revealed that the mRNA expression levels of *Trpa1, Trpv1*, and *Trpv4* were significantly increased in the DRGs of the dry skin-induced chronic itch model. Additionally, the TRPA1 expression in the dry skin was reduced by the Zn^2+^ chelators. Together, TRPA1 may be a direct molecular target for Zn^2+^-mediating acute and chronic itch.

In addition, our pharmacological assays demonstrated that histamine, mast cells, capsaicin-sensitive C-fibers, and opioid receptors were involved in the acute itch induced by Zn^2+^ in the mice. Besides the direct activation of primary sensory neurons, our results suggested that skin mast cells may also be a potential target for exogenous Zn^2+^. We also observed that systemic administration of Zn^2+^ chelator decreased the compound 48/80-induced itch in mice. Given the previous study showed mast cells can release Zn^2+^ (Nakashima-Kaneda et al., 2013), our data suggested that Zn^2+^ released from mast cells may also contribute to compound 48/80-induced itch. Involvement of non-neuronal cells (e.g., mast cells and keratonocytes) in Zn^2+^-induced itch warrants further investigations.

### Activation of ERK Signaling Is Involved in Zn^2+^-Mediated Itch in Mice

Extracellular signal-regulated kinase activation is well-known for regulating pain signaling transmission (Ma and Quirion, 2005). Recent studies have shown that ERK activation in the DRGs also drives the development of chronic itch in mice (Zhao et al., 2013). ERK activation in the skin participated in histamine- or MGO-induced itch in mice (Chen et al., 2016; Cheng et al., 2019). We further demonstrated that ERK activation in the DRGs and skin was indeed involved in Zn^2+^-induced acute itch in mice. Under chronic itch conditions, after systemic administration of Zn^2+^ chelators in mice, the expression level of p-ERK in the DRG and the skin was significantly reduced. Moreover, the incubation of Zn^2+^ with HaCaT cells activated p-ERK *in vitro*. Thus, it indicated that endogenous Zn^2+^ may be involved in ERK signaling activation under chronic itch conditions. Our data suggested that targeting ERK signaling may be effective for anti-itch treatment.

### The Possible Role of Zn^2+^-Sensing GPR39 in Zn^2+^-Mediated Itch in Mice

Given GPR39 as a Zn^2+^-sensing GPCR, we predicted that GPR39 may also be involved in Zn^2+^-induced itch in mice. Unexpectedly, i.d. injection of a selective GPR39 agonist TC-G-1008 was not able to evoke scratching in mice, suggesting that activation GPR39 may not be sufficient for inducing acute itch in naïve mice. Consistently, the RT-PCR analysis showed that the expression levels of GPR39 were relatively lower than the skin and the spinal cord.

In contrast, i.d. injection of TC-G-1008 significantly increased the scratching behavior under dry skin-induced chronic itch condition in mice. Moreover, the mRNA expression levels of GPR39 significantly increased in the skin of dry skin mice, while the increased expression of GPR39 in the skin was abolished in systemic Zn^2+^ chelators-treated mice. After pre-incubation with ZnCl_2_ in the HaCaT cells, the mRNA expression of GPR39 was significantly upregulated after ZnCl_2_ treatment for 0.5 and 1 h. Furthermore, pre-incubation with Zn^2+^ in the HaCaT cells significantly upregulated the expression of proinflammatory and itch mediators, including IL-6, IL-33, and TSLP. Systemic treatment of Zn^2+^ chelators also abolished the upregulation of the expression of proinflammatory and pruritogenic mediators in the skin of mice. Therefore, we speculated that under chronic itch conditions, endogenous Zn^2+^ was sensed by GPR39 in the skin, and the activation of GPR39 may lead to the production or release of proinflammatory and pruritogenic mediators from the keratinocytes, which contributes to the development of chronic itch. The causal relationship between GPR39 activation and ERK phosphorylation in the skin under chronic itch conditions warrants further investigations. Nevertheless, previous studies demonstrated that GPR39 activation in the keratinocytes by zinc release from injury cells promotes epithelial repair, possibly through intracellular calcium and p-ERK signaling pathways (Sharir et al., 2010). This raised another interesting question whether extracellular Zn^2+^ sensed by GPR39 in the skin is involved in wound healing-induced itch or not (Xu et al., 2020).

In summary, we have demonstrated that TRPA1, GPR39, and p-ERK signals were involved in both Zn^2+^-mediated acute and chronic itch in mice. The chronic itch may cause Zn^2+^ dyshomeostasis in the DRGs, possibly by dysregulated expression of ZIPs and ZnTs. In conclusion, our results revealed novel mechanisms underlying the itch and provided strong evidence that targeting the Zn^2+^/TRPA1/GPR39/ERK signaling pathway may be a promising strategy for the management of acute and chronic itch.

## Data Availability Statement

The original contributions presented in the study are included in the article/Supplementary Material, further inquiries can be directed to the corresponding authors.

## Ethics Statement

The animal study was reviewed and approved by the Ethics Committee for the Use of Experimental Animals in Soochow University Animal Committee.

## Author Contributions

YH, Q-YF, D-NF, X-LW, Z-HW, J-TZ, W-JX, and G-KZ contributed to the work design, performed experiments, and analyzed and interpreted data from all the experiments. Animal behavior experiments were performed by YH, Q-YF, and D-NF. Molecular biology experiments were performed by X-LW, Z-HW, J-TZ, W-JX, and G-KZ. YH, Q-YF, L-HC, and TL wrote and completed the manuscript. All authors critically revised and approved the final manuscript and agreed to take the responsibility for all aspects of the study.

## Funding

This study was supported by the National Natural Science Foundation of China (81870874 and 82171229 to TL; 81803307 to L-HC).

## Conflict of Interest

The authors declare that the research was conducted in the absence of any commercial or financial relationships that could be construed as a potential conflict of interest.

## Publisher's Note

All claims expressed in this article are solely those of the authors and do not necessarily represent those of their affiliated organizations, or those of the publisher, the editors and the reviewers. Any product that may be evaluated in this article, or claim that may be made by its manufacturer, is not guaranteed or endorsed by the publisher.
