# Supplementary material for: The Role of Transient Receptor Potential A1 and G Protein-Coupled Receptor 39 in Zinc-Mediated Acute and Chronic Itch in Mice
Source: Front Mol Neurosci. 2022 Jan 12;14:768731. doi: 10.3389/fnmol.2021.768731 (PMC8790520; doi:10.3389/fnmol.2021.768731)
Supplement: Supplementary file 1 [file Data_Sheet_1.docx]

Supplementary Material

**Table S1. Summary of statistical analysis used in this study**

| Figure | Test used | *n* value | Comparison | F value or t value | *P* value |
| --- | --- | --- | --- | --- | --- |
| Fig.1A | one-way ANOVA | *n* = 6-7 | (1 - 150 mM) ZnCl_2_ vs Saline | F_(6, 36)_ = 90.11 | *P <* 0.0001 |
| Fig.1A | unpaired Student’s *t*-test | *n* = 6 | 50 mM ZnCl_2_ vs. 150 mM ZnCl_2_. | t_10_ = 4.714. | *P* = 0.0008 |
| Fig.1B | one-way ANOVA | *n* = 6-8 | (0.3 - 150 mM) Zn(CH_3_COO)_2_ vs Saline | F_(7, 42)_ = 40.73 | *P* < 0.0001 |
| Fig.1B | unpaired Student’s *t*-test | *n* = 6 | 50 mM Zn(CH_3_COO)_2_ vs 150 mM Zn(CH_3_COO)_2_ | t_10_ = 2.283 | *P* = 0.0456 |
| Fig.1C | one-way ANOVA | *n* = 6 | (1 - 150 mM) ZnSO_4_ vs Saline | F_(4,25)_ = 70.36; | *P* < 0.0001; |
| Fig.1G | unpaired Student’s *t*-test | *n* = 6 | 10 μg Capsaicin vs Saline | t_10_ = 10.40 | *P* < 0.0001 |
| Fig.1H | unpaired Student’s *t*-test | *n* = 6 | 10 μg Capsaicin vs Saline | t_10_ = 1.515 | *P* = 0.1607 |
| Fig.1I | one-way ANOVA | *n* = 6 | (3 mM, 30 mM ) ZnCl_2_ vs Saline | F_(2, 15)_ = 2.154 | *P* = 0.1506 |
| Fig.1J | one-way ANOVA | *n* = 6-8 | (3 mM, 30 mM ) ZnCl_2_ vs Saline | F_(2, 17)_ = 53.36 | *P* < 0.0001 |
| Fig.2A | unpaired Student’s *t*-test | *n* = 6 | 300 μg Chlorpheniramine vs Saline | t_10_ = 3.444 | *P* = 0.0063 |
| Fig.2B | unpaired Student’s *t*-test | *n* = 6 | 300 μg Chlorpheniramine vs Saline | t_10_ = 3.410 | *P* = 0.0067 |
| Fig.2C | unpaired Student’s *t*-test | *n* = 6 | 3 mM ZnCl_2_ vs Saline | t_10_ = 2.241 | *P* = 0.3724 |
| Fig.2D | unpaired Student’s *t*-test | *n* = 6 | 3 mM ZnCl_2_ vs Saline | t_10_ = 2.241 | *P* = 0.3724 |
| Fig.2E | unpaired Student’s *t*-test | *n* = 7-8 | 3 mM ZnCl_2_ vs Saline | t_13_ = 5.553 | *P* < 0.0001 |
| Fig.2F | unpaired Student’s *t*-test | *n* = 7-8 | Compound 48/80 vs Saline | t_13_ = 5.621 | *P* < 0.0001 |
| Fig.2G | unpaired Student’s *t*-test | *n* = 6 | 1 mg/g Morphine vs Saline | t_10_ = 0.4009 | *P* = 0.6969 |
| Fig.2G | unpaired Student’s *t*-test | *n* = 6 | 1 mg/g Naloxone vs Saline | t_10_ = 6.540 | *P* < 0.0001 |
| Fig.2H | unpaired Student’s *t*-test | *n* = 6 | RTX vs Saline | t_10_ = 20.04 | *P* < 0.0001; |
| Fig.2I | unpaired Student’s *t*-test | *n* = 6 | RTX vs Saline | t_10_ = 8.785 | *P* < 0.0001 |
| Fig.2J | unpaired Student’s *t*-test | *n* = 8 | (50 mM ) ZnCl_2_ vs Saline | t_14_ = 1.408, | P = 0.1808 |
| Fig.3A | one-way ANOVA | *n* = 6-9 | (5 nmol, 15 nmol) Ruthenium Red vs Vehicle | F_(2, 19)_ = 30.62 | *P* < 0.0001 |
| Fig.3B | one-way ANOVA | *n* = 6 | (5 nmol, 15 nmol) Ruthenium Red vs Vehicle | F_(2, 15)_ = 47.24 | P < 0.0001 |
| Fig.3C | unpaired Student’s *t*-test | *n* = 6 | 50 μg A967079 vs Vehicle | t_10_ = 7.871 | *P* <0.0001 |
| Fig.3D | unpaired Student’s *t*-test | *n* = 6 | 50 μg A967079 vs Vehicle | t_10_ = 5.843 | *P* = 0.0002 |
| Fig.3E | one-way ANOVA | *n* = 6 | 50 μg HC030031 vs Vehicle  100 μg HC030031 vs Vehicle | F_(2, 15)_ = 17.49 | *P* = 0.0001 |
| Fig.3F | one-way ANOVA | *n* = 6 | 50 μg HC030031 vs Vehicle  100 μg HC030031 vs Vehicle | F_(2, 15)_ = 69.57 | *P* < 0.0001 |
| Fig.3G | unpaired Student’s *t*-test | *n* = 6 | 50 μg Capsazepine vs Vehicle | t_10_ = 0.2132 | *P* = 0.8354 |
| Fig.3H | unpaired Student’s *t*-test | *n* = 6 | 50 μg Capsazepine vs Vehicle | t_10_ = 0.1744 | *P* = 0.8650 |
| Fig.3I | unpaired Student’s *t*-test | *n* = 6-8 | 50 μg HC067047 vs Vehicle | t_12_ = 0.7413 | *P* = 0.4728 |
| Fig.3J | unpaired Student’s *t*-test | *n* = 6-8 | 50 μg HC067047 vs Vehicle | t_10_ = 1.316 | *P* = 0.2176 |
| Fig.4A | unpaired Student’s *t*-test | *n* = 6 | *Trpa1*^-/-^ vs WT | t_10_ = 6.054 | *P* = 0.0001 |
| Fig.4B | unpaired Student’s *t*-test | *n* = 6 | *Trpa1^-/-^* vs WT | t_10_ = 4.651 | *P* = 0.0009 |
| Fig.4C | unpaired Student’s *t*-test | *n* = 6 | *Trpv1*^-/-^ vs WT | t_10_ = 0.2377 | *P* = 0.8169 |
| Fig.4D | unpaired Student’s *t*-test | *n* = 6 | *Trpv1*^-/-^ vs WT | t_10_ = 0.4894 | *P* = 0.6351 |
| Fig.4E | unpaired Student’s *t*-test | *n* = 6-7 | *Trpv4*^-/-^ vs WT | t_11_ = 0.4754 | *P* = 0.6438 |
| Fig.4F | unpaired Student’s *t*-test | *n* = 6-7 | *Trpv4*^-/-^ vs WT | t_11_ = 0.3608 | *P* = 0.7251 |
| Fig.5A | one-way ANOVA | *n* = 6 | (1 mg/kg, 10 mg/kg) TPEN vs Vehicle | F_(2, 15)_ = 7.133 | *P* = 0.0067 |
| Fig.5B | one-way ANOVA | *n* = 6 | (1 mg/kg、3 mg/kg、10 mg/kg) TPEN vs Vehicle | F_(3, 20)_ = 22.85 | *P* < 0.0001 |
| Fig.5C | one-way ANOVA | *n* = 6 | (1 mg/kg、3 mg/kg、10 mg/kg) TPEN vs Vehicle | F_(3, 20)_ = 20.66 | *P* < 0.0001 |
| Fig.5D | one-way ANOVA | *n* = 6 | (5 mg/kg, 10 mg/kg) Pyrithione vs Vehicle | F_(2, 15)_ = 19.79 | *P* < 0.0001 |
| Fig.5E | one-way ANOVA | *n* = 6 | (5 mg/kg, 10 mg/kg) Pyrithione vs Vehicle | F_(2, 15)_ = 11.13 | *P* = 0.0011 |
| Fig.5F | one-way ANOVA | *n* = 6 | (5 mg/kg, 10 mg/kg) Pyrithione vs Vehicle | F_(2, 15)_ = 12.24 | *P* = 0.0007 |
| Fig.5G | one-way ANOVA | *n* = 6 | (5 mg/kg, 10 mg/kg) Clioquinol vs Vehicle | F_(2, 15)_ = 43.70 | *P* < 0.0001 |
| Fig.5H | one-way ANOVA | *n* = 6 | (5 mg/kg, 10 mg/kg) Clioquinol vs Vehicle | F_(2, 15)_ = 33.69 | *P* < 0.0001 |
| Fig.5I | one-way ANOVA | *n* = 6 | (5 mg/kg, 10 mg/kg) Clioquinol vs Vehicle | F_(2, 15)_ = 13.89 | *P* = 0.0004 |
| Fig.5J | unpaired Student’s *t*-test | *n* = 6 | (10 mg/kg) TPEN vs Vehicle | t_10_ = 5.802 | *P* = 0.0002 |
| Fig.5J | unpaired Student’s *t*-test | *n* = 6 | (10 mg/kg) Pyrithione vs Vehicle | t_10_ = 1.274 | *P* = 0.2314 |
| Fig.5J | unpaired Student’s *t*-test | *n* = 6 | (10 mg/kg) Clioquinol vs Vehicle | t_10_ = 2.356 | *P* = 0.0402 |
| Fig.5K | unpaired Student’s *t*-test | *n* = 7 | (10 mg/kg) TPEN vs Vehicle | t_12_ = 0.6283 | *P* = 0.5416 |
| Fig.5K | unpaired Student’s *t*-test | *n* = 7 | (10 mg/kg) Pyrithione vs Vehicle | t_12_ = 0.4426 | *P* = 0.6659 |
| Fig.5K | unpaired Student’s *t*-test | *n* = 7 | (10 mg/kg) Clioquinol vs Vehicle | t_12_ = 1.996 | *P* = 0.0691 |
| Fig.6B | two-way ANOVA | *n* = 6-10 | AEW + Vehicle vs Ctrl AEW + 3 mg/kg TPEN vs AEW + Vehicle  AEW + 5 mg/kg Pyrithione vs AEW + Vehicle  AEW + 5 mg/kg Clioquinol vs AEW + Vehicle | Time: F_(4, 160)_ = 46.59;  Group: F_(4, 160)_ = 53.17;  Interaction: F_(16, 160)_ = 10.40. | *P* < 0.0001;  *P* < 0.0001;  *P* < 0.0001. |
| Fig.6C | unpaired Student’s *t*-test | *n* = 5 | *Trpa1*: AEW vs Ctrl | t_8_ = 4.798 | *P* = 0.0014 |
| Fig.6C | unpaired Student’s *t*-test | *n* = 5 | *Trpv1*: AEW vs Ctrl | t_8_ = 3.258 | *P* = 0.0116 |
| Fig.6C | unpaired Student’s *t*-test | *n* = 5 | *Trpv4*: AEW vs Ctrl | t_8_ = 4.297 | *P* = 0.0026 |
| Fig.6E | unpaired Student’s *t*-test | *n* = 4 | AEW + 5 mg/kg Clioquinol vs AEW + Vehicle | t_4_ = 11.43, | *P* = 0.0003 |
| Fig.6F | unpaired Student’s *t*-test | *n* = 5 | AEW + 3 mg/kg TPEN vs AEW + Vehicle | t_8_ = 0.09126 | P = 0.9295 |
| Fig.6F | unpaired Student’s *t*-test | *n* = 5 | AEW + 5 mg/kg Pyrithione vs AEW + Vehicle | t_8_ = 2.941 | P = 0.0187 |
| Fig.6F | unpaired Student’s *t*-test | *n* = 5 | AEW + 5 mg/kg Clioquinol vs AEW + Vehicle | t_8_ = 6.355 | P = 0.0002 |
| Fig.7D | unpaired Student’s *t*-test | *n* = 5 | *Slc39a1*: AEW vs Ctrl | t_8_ = 2.947 | *P* = 0.0185 |
| Fig.7D | unpaired Student’s *t*-test | *n* = 5 | *Slc39a2*: AEW vs Ctrl | t_8_ = 4.494 | *P* = 0.0020 |
| Fig.7D | unpaired Student’s *t*-test | *n* = 5 | *Slc39a4*: AEW vs Ctrl | t_8_ = 4.677 | *P* = 0.0016 |
| Fig.7D | unpaired Student’s *t*-test | *n* = 5 | *Slc39a6*: AEW vs Ctrl | t_8_ = 2.807 | *P* = 0.0230 |
| Fig.7D | unpaired Student’s *t*-test | *n* = 5 | *Slc39a9*: AEW vs Ctrl | t_8_ = 2.317 | *P* = 0.0491 |
| Fig.7D | unpaired Student’s *t*-test | *n* = 5 | *Slc39a3*: AEW vs Ctrl | t_8_ = 4.494 | *P* = 0.0020 |
| Fig.7D | unpaired Student’s *t*-test | *n* = 5 | *Slc39a5*: AEW vs Ctrl | t_8_ = 3.499 | *P* = 0.0081 |
| Fig.7D | unpaired Student’s *t*-test | *n* = 5 | *Slc39a7*: AEW vs Ctrl | t_8_ = 6.393 | *P* = 0.0002 |
| Fig.7D | unpaired Student’s *t*-test | *n* = 5 | *Slc39a8*: AEW vs Ctrl | t_8_ = 3.502 | *P* = 0.0081 |
| Fig.7D | unpaired Student’s *t*-test | *n* = 5 | *Slc39a10*: AEW vs Ctrl | t_8_ = 3.273 | *P* = 0.0113 |
| Fig.7D | unpaired Student’s *t*-test | *n* = 5 | *Slc39a11*: AEW vs Ctrl | t_8_ = 3.219 | *P* = 0.0123 |
| Fig.7D | unpaired Student’s *t*-test | *n* = 5 | *Slc39a12*: AEW vs Ctrl | t_8_ = 6.940 | *P* = 0.0001 |
| Fig.7D | unpaired Student’s *t*-test | *n* = 5 | *Slc39a13*: AEW vs Ctrl | t_8_ = 7.493 | *P* < 0.0001 |
| Fig.7D | unpaired Student’s *t*-test | *n* = 5 | *Slc39a14*: AEW vs Ctrl | t_8_ = 4.677 | P = 0.0016 |
| Fig.7D | unpaired Student’s *t*-test | *n* = 5 | *Slc30a1*: AEW vs Ctrl | t_8_ = 6.096 | *P* = 0.0003 |
| Fig.7E | unpaired Student’s *t*-test | *n* = 5 | *Slc30a4*: AEW vs Ctrl | t_8_ = 4.638 | *P* = 0.0017 |
| Fig.7E | unpaired Student’s *t*-test | *n* = 5 | *Slc30a5*: AEW vs Ctrl | t_8_ = 4.270 | *P* = 0.0027 |
| Fig.7E | unpaired Student’s *t*-test | *n* = 5 | *Slc30a2*: AEW vs Ctrl | t_8_ = 3.215 | *P* = 0.0123 |
| Fig.7E | unpaired Student’s *t*-test | *n* = 5 | *Slc30a3*: AEW vs Ctrl | t_8_ = 3.038 | *P* = 0.0161 |
| Fig.7E | unpaired Student’s *t*-test | *n* = 5 | *Slc30a6*: AEW vs Ctrl | t_8_ = 2.743, | P = 0.0253 |
| Fig.8A | unpaired Student’s *t*-test | *n* = 4 | 10 min vs Ctrl | t_6_ = 9.007 | *P* = 0.0001 |
| Fig.8A | unpaired Student’s *t*-test | *n* = 4 | 30 min vs Ctrl | t_6_ = 6.185 | *P* = 0.0008 |
| Fig.8B | unpaired Student’s *t*-test | *n* = 4 | 10 min vs Ctrl | t_6_ = 3.232 | *P* = 0.0179 |
| Fig.8B | unpaired Student’s *t*-test | *n* = 4 | 30 min vs Ctrl | t_6_ = 5.898 | *P* = 0.0011 |
| Fig.8C | unpaired Student’s *t*-test | *n* = 6 | U0126 vs Saline | t_10_ = 4.237 | *P* = 0.0017 |
| Fig.8D | unpaired Student’s *t*-test | *n* = 4 | AEW + Vehicle vs Ctrl | t_6_ = 10.54 | *P* < 0.0001 |
| Fig.8D | unpaired Student’s *t*-test | *n* = 4 | AEW + TPEN vs AEW + Vehicle | t_6_ = 8.165 | *P* = 0.0002 |
| Fig.8D | unpaired Student’s *t*-test | *n* = 4 | AEW + Pyrithione vs AEW + Vehicle | t_6_ = 5.272 | *P* = 0.0019 |
| Fig.8D | unpaired Student’s *t*-test | *n* = 4 | AEW + Clioquinol vs AEW + Vehicle | t_6_ = 7.286 | *P* = 0.0003 |
| Fig.8E | unpaired Student’s *t*-test | *n* = 4 | AEW + Vehicle vs Ctrl | t_6_ = 4.488 | *P* = 0.0042 |
| Fig.8E | unpaired Student’s *t*-test | *n* = 4 | AEW + TPEN vs AEW + Vehicle | t_6_ = 3.839 | *P* = 0.0086 |
| Fig.8E | unpaired Student’s *t*-test | *n* = 4 | AEW + Pyrithione vs AEW + Vehicle | t_6_ = 3.852 | *P* = 0.0084 |
| Fig.8E | unpaired Student’s *t*-test | *n* = 4 | AEW + Clioquinol vs AEW + Vehicle | t_6_ = 3.872 | *P* = 0.0082 |
| Fig.9B | one-way ANOVA | *n* = 6-8 | (10-100 μg) TC-G-1008 vs Saline | F_(4, 29)_ = 2.043 | *P* = 0.1145 |
| Fig.9C | two-way ANOVA | *n* = 8 | AEW + Vehicle vs Ctrl  AEW + TC-G-1008 vs AEW + Vehicle | Time: F_(4, 105)_ = 58.55;  Group: F_(2, 105)_ = 96.31;  Interaction: F_(8, 105)_ = 16.69. | *P* < 0.0001;  *P* < 0.0001;  *P* < 0.0001. |
| Fig.9D | unpaired Student’s *t*-test | *n* = 5 | AEW + Vehicle vs Ctrl | GPR39: t_8_ = 6.055;  IL-6: t_8_ = 5.267;  IL-33: t_8_ = 6.182;  TSLP: t_8_ = 2.317. | *P* = 0.0003;  *P* = 0.0008;  *P* = 0.0003;  *P* = 0.0492. |
| Fig.9D | unpaired Student’s *t*-test | *n* = 5 | AEW + TPEN vs AEW + Vehicle | GPR39: t_8_ = 3.495;  IL-6: t_8_ = 5.277;  IL-33: t_8_ = 2.892;  TSLP: t_8_ = 2.517. | *P* = 0.0081;  *P* = 0.0007;  *P* = 0.0201;  *P* = 0.0360. |
| Fig.9D | unpaired Student’s *t*-test | *n* = 5 | AEW + Pyrithione vs AEW + Vehicle | GPR39: t_8_ = 6.611;  IL-6: t_8_ = 8.837;  IL-33: t_8_ = 5.312;  TSLP: t_8_ = 4.246. | *P* = 0.0002;  *P* < 0.0001;  *P* = 0.0007;  *P* = 0.0028. |
| Fig.9D | unpaired Student’s *t*-test | *n* = 5 | AEW + Clioquinol vs AEW + Vehicle | GPR39: t_8_ = 3.243;  IL-6: t_8_=2.859;  IL-33: t_8_ = 3.475 ;  TSLP: t_8_ = 3.547. | *P* = 0.0118;  *P* = 0.0212;  *P* = 0.0084;  *P* = 0.0075. |
| Fig.9E | unpaired Student’s *t*-test | *n* = 5-6 | 0.5 h vs Ctrl | t_9_ = 5.055 | *P* = 0.0007 |
| Fig.9E | unpaired Student’s *t*-test | *n* = 5-6 | 1 h vs Ctrl | t_9_ = 2.516 | *P* = 0.0330 |
| Fig.9E | unpaired Student’s *t*-test | *n* = 6 | 2 h vs Ctrl | t_10_ = 0.1994 | *P* = 0.8459 |
| Fig.9F | unpaired Student’s *t*-test | *n* = 3 | 0.5 h vs Ctrl | t_4_ = 3.718 | *P* = 0.0205 |
| Fig.9F | unpaired Student’s *t*-test | *n* = 3 | 1 h vs Ctrl | t_4_ = 2.271 | *P* = 0.0857 |
| Fig.9F | unpaired Student’s *t*-test | *n* = 3 | 2 h vs Ctrl | t_4_ = 1.220 | *P* = 0.2895 |

**Table S2. The following primers used in this study were synthesized by Genewiz.**

| Primer | sequence（5’ to 3’） |
| --- | --- |
| GAPDH-Mouse-F | GAAGGTCGGTGTGAACGGAT |
| GAPDH-Mouse-R | AATCTCCACTTTGCCACTGC |
| TRPV1-Mouse-F | ACCACGGCTGCTTACTATCG |
| TRPV1-Mouse-R | GCTGGAATCCTCGGGTGTAG |
| TRPV4-Mouse-F | TCCTTCTACATCAACGTGGTCTC |
| TRPV4 -Mouse-R | CACTGTGGTCCGGTAAGGGTAG |
| TRPA1 -Mouse-F | GGAAATACCCCACTGCATTGT |
| TRPA1 -Mouse-R | CAGCTATGTGAAGGGGTGACA |
| SLC39A1 -Mouse-F | TGCTTGTGTCCTGGTCTTCTC |
| SLC39A1 -Mouse-R | ACAGGCTGACTGCCAGAATG |
| SLC39A2 -Mouse-F | AGCCGCTGGCACGTTTTTATA |
| SLC39A2 -Mouse-R | ACCCAGCAGCCACACAGCTA |
| SLC39A3-Mouse-F | TGTCAGCTTCTCCTATGGCTTGT |
| SLC39A3 -Mouse-R | GGATCCCGCCTGCACTAATA |
| SLC39A4 -Mouse-F | TCTGAGAAAGATGGGCCTTGTAG |
| SLC39A4 -Mouse-R | TGTTTGGACTGTCGGAGATTG |
| SLC39A5 -Mouse-F | TCCAGTGGCCTCAGCACTA |
| SLC39A5 -Mouse-R | AGCAGCTTCCGAAAGGATAA |
| SLC39A6 -Mouse-F | ACAGGGATATTCATCGGGCATTA |
| SLC39A6-Mouse-R | AACCAGAGCGACATACATGAACAAG |
| SLC39A7-Mouse-F | TGCTGCCTGAGCTATTGAGAGA |
| SLC39A7-Mouse-R | AATCAGTACCATCATGGCAACAC |
| SLC39A8-Mouse-F | AACAATTGCCTGGATGATCAC |
| SLC39A8-Mouse-R | CAAAGTACAAGATGCCCCAATC |
| SLC39A9-Mouse-F | TAAGAGCAGTAAAGAAGCCCTTTCA |
| SLC39A9-Mouse-R | ACGGTGGCAACATAAAGAAATC |
| SLC39A10-Mouse-F | GCCCTTCACCAGAGACCAATAA |
| SLC39A10-Mouse-R | CCTCCTGACCTTCACTGACTTCA |
| SLC39A11-Mouse-F | CTCACCTGGGTGCTACAGAAGAC |
| SLC39A11-Mouse-R | CAATGCAGGGTCCAAGTTCA |
| SLC39A12-Mouse-F | AATGTGCCAGCCTCCAACA |
| SLC39A12-Mouse-R | TTACTAGGCCATCTGCAAAATTGTG |
| SLC39A13-Mouse-F | TGCTTGCCAACACCATAGACAAC |
| SLC39A13-Mouse-R | CAGAAGCCCGATCTTTTTGC |
| SLC39A14-Mouse-F | TCTGCCAGGAGGATGAGAAG |
| SLC39A14-Mouse-R | ATAATGGAGAAGCCGGTTAGG |
| SLC30A1-Mouse-F | CCTGGGCTTCRTCTCTAGATTG |
| SLC30A1-Mouse-R | TGTCTTGGAAAGGTTGTTCTG |
| SLC30A2-Mouse-F | TGAGGTGCCTACTTGCTCCT |
| SLC30A2-Mouse-R | TTCCAGCTGCTCCCTGTACT |
| SLC30A3-Mouse-F | CCTGGTCTCTGTACTGTCCATCT |
| SLC30A3-Mouse-R | GATCACGAACAGCTGTGAAGTC |
| SLC30A4-Mouse-F | CCATCCTCATCTACTTCAAGCC |
| SLC30A4-Mouse-R | CAGAGGCAACATGGTAAGTGAG |
| SLC30A5-Mouse-F | GGAAGCGCCTCAAATCTATGCT |
| SLC30A5-Mouse-R | TACATTCAAAATGGCTTGGCACA |
| SLC30A6-Mouse-F | GATAGCCTGGCAGTGAGAGCTG |
| SLC30A6-Mouse-R | TACATTCAAAATGGCTTGGCACA |
| SLC30A7-Mouse-F | CACACCAGGAGAGCACTGAA |
| SLC30A7-Mouse-R | TCAGCCCTCCAACTGAGACT |
| SLC30A8-Mouse-F | TGGCAGCCAACATTGTACTAAC |
| SLC30A8-Mouse-R | TCTCATGCTGTGGATTCCTAGA |
| SLC30A9-Mouse-F | CTTTACTGTGTTCTTGAGGTC |
| SLC30A9-Mouse-R | GGGTCGCTCTCCAGGAGTTCA |
| GPR39-Mouse-F | CTGATCATCTTTGTGGTAGGCA |
| GPR39-Mouse-R | ATGGTCTGTCTTCTTGCTGTCC |
| IL-6-Mouse-F | ACTTCACAAGTCCGGAGAGG |
| IL-6-Mouse-R | TGCAAGTGCATCATCGTTGT |
| IL-33-Mouse-F | GATGGGAAGAAGCTGATGGTG |
| IL-33-Mouse-R | TTGTGAAGGACGAAGAAGGC |
| TSLP-Mouse-F | ACGGATGGGGCTAACTTACAA |
| TSLP-Mouse-R | AGTCCTCGATTTGCTCGAACT |
| GAPDH-Human-F | AAGGTGAAGGTCGGAGTCAA |
| GAPDH-Human-R | AATGAAGGGGTCATTGATGG |
| GPR39-Human-F | CTTGCTGTTTGCCATGGGTACTGA |
| GPR39-Human-R | GGCCAATGTCACAACAATCAGCCT |
